# Wolves and Dogs May Rely on Non-numerical Cues in Quantity Discrimination Tasks When Given the Choice

**DOI:** 10.3389/fpsyg.2020.573317

**Published:** 2020-09-11

**Authors:** Dániel Rivas-Blanco, Ina-Maria Pohl, Rachel Dale, Marianne Theres Elisabeth Heberlein, Friederike Range

**Affiliations:** ^1^Domestication Lab, Department of Interdisciplinary Life Sciences, Konrad Lorenz Institute of Ethology, University of Veterinary Medicine Vienna, Vienna, Austria; ^2^Clever Dog Lab, Comparative Cognition, Messerli Research Institute, University of Veterinary Medicine Vienna, Medical University of Vienna, University of Vienna, Vienna, Austria; ^3^Wolf Science Center, Ernstbrunn, Austria

**Keywords:** numerical competence, quantity discrimination, Weber’s law, non-numerical information, wolves, dogs

## Abstract

A wide array of species throughout the animal kingdom has shown the ability to distinguish between quantities. Aside from being important for optimal foraging decisions, this ability seems to also be of great relevance in group-living animals as it allows them to inform their decisions regarding engagement in between-group conflicts based on the size of competing groups. However, it is often unclear whether these animals rely on numerical information alone to make these decisions or whether they employ other cues that may covary with the differences in quantity. In this study, we used a touch screen paradigm to investigate the quantity discrimination abilities of two closely related group-living species, wolves and dogs, using a simultaneous visual presentation paradigm. Both species were able to successfully distinguish between stimuli of different quantities up to 32 items and ratios up to 0.80, and their results were in accordance with Weber’s law (which predicts worse performances at higher ratios). However, our controls showed that both wolves and dogs may have used continuous, non-numerical cues, such as size and shape of the stimuli, in conjunction with the numerical information to solve this task. In line with this possibility, dogs’ performance greatly exceeded that which they had shown in other numerical competence paradigms. We discuss the implications these results may have on these species’ underlying biases and numerical capabilities, as well as how our paradigm may have affected the animals’ ability to solve the task.

## Introduction

The ability to discriminate different quantities proves to be a very useful tool for humans and animals alike. For example, assessing which areas have the most food and mating opportunities, as well as the fewest predators or competitors, often requires at least basic quantity judgment skills. Therefore, it is not surprising that a large array of species have demonstrated numerical competence to a certain extent ranging from insects (Reznikova and Ryabko, 2011; Gatto and Carlesso, 2019), to cuttlefish (Yang and Chiao, 2016); to vertebrates such as fish (Agrillo et al., 2010; Gómez-Laplaza and Gerlai, 2011; Potrich et al., 2015), amphibians (Krusche et al., 2010; Lucon-Xiccato et al., 2018), lizards (Miletto Petrazzini et al., 2018), tortoises (Gazzola et al., 2018), birds (Hunt et al., 2008; Ditz and Nieder, 2016; Kelly, 2016), and mammals (Hauser et al., 2003; Jordan and Brannon, 2006; Beran et al., 2008; Pisa and Agrillo, 2009; Irie and Hasegawa, 2012; Vonk and Beran, 2012).

Species that live in groups and defend home ranges benefit especially from possessing numerical abilities, as they provide them with useful information to decide whether to engage in inter-group conflict during territory defense (Manson and Wrangham, 1991). In a seminal study, McComb et al. (1994) found that lionesses were less likely to approach an audio playback of three unfamiliar female lions than a playback of a single female and groups of two were less likely to approach the speaker than groups of three or more. This was the first of a number of studies to show that group-living animals are able to assess resource-holding potential (i.e., the ability to acquire or defend resources; Parker, 1974) on the basis of relative group size (e.g., chimpanzees: Wilson et al., 2001; hyenas: Benson-Amram et al., 2011; howler monkeys: Kitchen, 2004; banded mongooses: Furrer et al., 2011; dogs: Bonanni et al., 2010, 2011; and wolves: Harrington and Mech, 1979; Cassidy et al., 2015).

Although such studies provide great insights into the natural behavior of the animals, they are unable to identify the precise mechanisms that the animals utilize to make quantity judgments. For example, it is possible that animals rely on perceptual cues, such as the cumulative size or the density of the stimuli, to assess quantity, rather than using the absolute number of items presented. Experimental studies have therefore endeavored to control for these cues. For example, Jordan and Brannon (2006) used a delayed match-to-sample paradigm whereby rhesus macaques were trained to choose between two options, one of which (the correct choice) contained the same number of items as a previously demonstrated sample stimulus. They systematically tested for the influence of element size, cumulative surface area, and density; they found that monkeys do base their choices on number, regardless of these continuous cues (see also Beran et al., 2008; Hunt et al., 2008; Gross et al., 2009; and Agrillo et al., 2011 for similar examples).

There are several factors that affect an animal’s capability to discern between quantities. First and foremost, performance conforms to Weber’s law in most species. This law states that the capacity to discriminate between two quantities increases as the ratio between them decreases (i.e., animals should perform better when discriminating between 2 and 8 items –a ratio of 0.25– than between 6 and 8 –a ratio of 0.75; Agrillo and Bisazza, 2014). Other factors that seem to affect performance in numerical tasks are the magnitude (defined as the total amount across both quantities; e.g., the magnitude of 6 vs. 8 would be 14) and the disparity (the absolute difference between the two quantities; e.g., the disparity of 6 vs. 8 would be 2). Thus, as magnitude *increases*, performance *decreases*; and the same happens when disparity *decreases* or the ratio becomes *more even* (Irie and Hasegawa, 2012).

Additionally, these factors seem to compound in such a manner that the effect of the ratio on performance is enhanced when higher magnitudes are presented. Hence, the difference in performance between smaller and larger ratios is less pronounced or even absent when lower numbers are used than when higher ones are (e.g., a high ratio like 0.75 can be discriminated at low magnitudes –such as 3 vs. 4– but not at high ones –like 12 vs. 16). This effect has been found in several species, including guppies (Agrillo et al., 2012), New Zealand robins (Hunt et al., 2008), domestic chicks (Rugani et al., 2008), and humans (Agrillo et al., 2012).

Interestingly, although magnitude does increase the effect of the ratio, absolute upper limits for magnitude do not seem to apply in most cases. As long as the ratio is low enough for them to distinguish, most vertebrates seem to discriminate quantities regardless of the total amount of items present (Agrillo et al., 2010, 2012; Beran and Parrish, 2016).

The effect of ratio and magnitude on performance in numerical tasks has led some researchers to believe that the processing of the different parts of the number range may be regulated by two distinct systems: one that can process only small quantities, but in a precise and fast manner (known as the “object-file” system) and the other that has seemingly no upper limit in magnitude, but is subject to a limit in ratio (known as the analog magnitude system) (Feigenson et al., 2004; Agrillo et al., 2012).

The numerical capabilities of a vast number of species have been tested within different settings and through several distinct paradigms. Two closely related species that have been investigated both under field and lab conditions are wolves and dogs.

Although both wolves and dogs have shown to be able to distinguish between different quantities in intergroup-conflicts under natural conditions (Harrington and Mech, 1979; Bonanni et al., 2010, 2011; Cassidy et al., 2015), their response in controlled lab experiments markedly differs. The first experimental studies in dogs revealed mixed results, with a preferential looking time task finding that dogs can discriminate between 1 vs. 2 and 2 vs. 3 (West and Young, 2002), whereas in a food choice task the dogs seemed unable to distinguish between two amounts of food differing only by one piece, regardless of the ratio (Ward and Smuts, 2007). In a following study using a sequential presentation paradigm where pieces of food were dropped one-by-one into a bowl, the dogs were unsuccessful in all pairings but 1 vs. 0 (Macpherson and Roberts, 2013). However, Macpherson and Roberts (2013) also piloted a paradigm with one dog whereby non-food stimuli were simultaneously presented on two boards, of which the dog could select one. In this setup, the dog was successful on a variety of pairings using numbers from 0 to 9, suggesting factors such as training, presentation of the stimuli, and food visibility may have affected dogs’ performance in previous studies.

So far, wolves have shown greater success in quantity discrimination tasks. Utrata et al. (2012) performed a study in which two sets of 1 to 4 pieces of food were inserted sequentially into two opaque tubes. The subjects could then choose one of the tubes, the larger set being considered the correct choice in all cases. The wolves were able to discriminate all pairs and were not affected by ratio (up to 0.75) with these low numbers. Crucially, the study controlled for the potential influence of the amount of time it took to insert different quantities of items. During these controls, the experimenter would insert additional stones into the tube with less food pieces so that the same number of items would be dropped on both sides, and thus they would require approximately the same amount of time to fill.

Wolves also outperformed dogs tested on the exact same paradigm (Range et al., 2014). The dogs were successful only on ratios up to 0.50, which suggests a potential difference between dogs and wolves in numerical competence. These results were reexamined by Miletto Petrazzini and Wynne (2017), who tested wolves and dogs by using the same quantity pairings but with a different method of stimuli presentation; they presented the food items simultaneously on two trays and allowed the animals to choose one of them. Their results corroborate those of Utrata et al. (2012) and Range et al. (2014), with wolves performing above chance on all pairs and dogs showing success only at ratios up to 0.50. Taken together, these studies suggest some differences in numerical discrimination capabilities between wolves and dogs and raise the question of whether they rely on the same information to make their quantity judgments.

The observed differences could come either as a result of the process of domestication, their social ecology, or a combination of both. On one hand, as Frank (1980) hypothesized, the domestication process may have reduced the effect of natural selection on dogs, leaving them with comparatively worse cognitive abilities. On the other, as proposed by Marshall-Pescini et al. (2017), differences in problem solving abilities between wolves and dogs may come as a result of adaptation to the niches they occupy. In this specific case, it is possible that assessing the numbers of competitors in inter-group conflicts is less important for dogs due to their more relaxed social dynamics (Mech and Boitani, 2010; Cafazzo et al., 2014) than wolves, for whom these conflicts inflict the highest natural mortality rate (Smith et al., 2015). Further, the feeding ecology of both species would also conform to this, as both pet and free-ranging dogs usually have easy access to food resources (Vanak and Gompper, 2009; Newsome et al., 2014) while wolves rely on hunting for sustenance and have a considerable probability of failure for each hunting bout (Mech and Boitani, 2010), which again makes quantity discrimination skills more relevant in wolves, as appropriately choosing the larger amount of food (e.g., the larger herds) may be vital for their survival.

All of the above studies are limited in terms of the magnitudes presented, their ability to control for some possibly confounding factors, and their ecological significance. In all these studies, magnitudes were low (with dogs being tested with numbers up to 9 and wolves up to 4), which may dampen the effect of the ratio on the subjects’ performance. Testing the animals with bigger numbers would likely not be feasible when using sequential paradigms as that would require them to memorize the quantities of two large sets of items, which could make it harder for them to draw comparisons. Moreover, the numbers used in these experiments may not be reflective of the natural conditions of the animals at least in the context of intergroup conflicts; dogs are facultatively social and usually live solitarily or in small packs, but they are also known to form groups of around 10 individuals; while wolf packs have an average size of around 5–8 individuals, and can go up to 42 (Font, 1987; Daniels and Bekoff, 1989; Bonanni et al., 2010; Cafazzo et al., 2014; Miklosi, 2015). Thus, testing them on numbers up to four may not make sense from a socio-ecological standpoint.

In addition to limited magnitude, the use of different quantities of food as the item to be counted may have been a confounding factor, as the subjects were rewarded even when they chose the smaller amount. Fernand et al. (2018) showed in a reverse-reward contingency task that dogs do not change their choices when selecting a specific stimulus gives them a smaller reward, possibly because they are rewarded regardless of their choice.

Thus, the aim of the current study is to expand on the literature by addressing some of the remaining questions on the topic of canid numerosity. We investigated dogs’ and wolves’ performance at discriminating quantities presented with different ratios and magnitudes, the latter of which were divided in two phases (with low and high numbers).

Both pet dogs and wolves raised in captivity were tested in this study. They were trained to select either a larger or smaller quantity of dots, presented simultaneously on a touch screen. They were then tested on both familiar (those used during the training) and novel pairs of quantities ranging from 1 to 8. The subjects that showed success in this stage were subsequently tested in a second phase using numbers ranging from 1 to 32. Using a touch screen to assess the quantity discrimination abilities of carnivores has been successfully done in the past (Vonk and Beran, 2012), and touch screens have also been used with canids prior to the current study (Range et al., 2008). Thus, we decided to use a touch screen paradigm, which allowed for greater standardization in the presentation of stimuli, avoidance of potential experimenter cues, removal of the possibly confounding effect of the presence of food during the choice, and the ability to control non-numerical cues in great detail. Thus, unlike in previous studies, the current study controlled for cumulative surface area and spacing and shape of the array of stimuli.

We predicted that dogs would be successful on ratios up to 0.50, but would drop in performance on higher ratios and, in the second stage, higher magnitudes. Furthermore, since the use of continuous cues is often favored over numerical ones (Xiong et al., 2018), we predicted that removing the ability to use the former would result in poorer overall performance when compared with previous studies. Although wolves have not shown a ratio effect to date, Weber’s law is predicted to have a greater influence on larger numbers (Lemmon, 1928; Moyer and Landauer, 1967), therefore, we predicted wolves would be successful on all ratios in the first stage and show reduced success on higher magnitudes and ratios in the second stage.

## Materials and Methods

### Experimental Set-Up

#### Apparatus and General Set-Up

**FIGURE 1:**
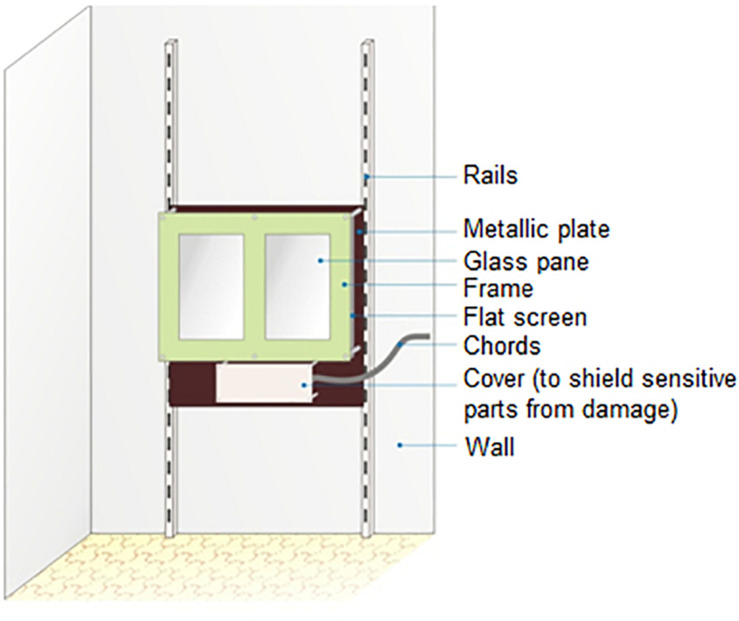
Schematic of the touch-screen used in this study.

We tested both the dogs and the wolves with a touch screen apparatus donated by Dietmar Schinnerl. It consisted of a flat screen fixed on a metal plate and mounted on a set of rails (see Figure 1); the height of the screen could be individually adjusted by moving the apparatus along the rails. Two separate acrylic glass panes were placed in front of the screen: one covering the left side and the other one the right side. Subjects would make their choices by pressing these glass panes with their muzzle, which activated the pressure-measuring elements to which the panes were connected. These pressure sensors were linked to a computer (which was running the program and recording the subjects’ inputs) and to a remote control that triggered a treat dispenser (“Manners Minder” by Premier), which would emit a high-pitched noise and release a treat in rewarded trials if the choice was correct (more information about the different types of trials below).

#### Stimuli Used

The stimuli were created and presented with an application written in “C#” (by Dietmar Schinnerl), based on “.NET Framework 2.0.” The stimuli consisted of randomized arrangements of different numbers of black dots on a white background. Stimuli pairs were divided between the ones with “small” numbers (1–8 dots in each stimulus) and those with “large” numbers (8–32 dots in each stimulus)^1^. The combinations of small and large numbers had similar ratios to each other except for one of the pairs presented to the dogs in which, due to an error, the combination of 16 vs. 32 dots (with a ratio of 0.50) was displayed instead as 16 vs. 22 dots (ratio of 0.73) for all but one of the tested subjects. A detailed account of all combinations throughout the training and test phases can be found in Table 1.

**TABLE 1:** Number pairs used in all phases, with their respective ratios.

**Phase 1**										
	**Training pairs**									
**Pairs**	1:8	1:7	1:6	1:5	1:4	2:8	2:7	2:6	3:8	
**Ratios**	0.13	0.14	0.17	0.20	0.25	0.25	0.29	0.33	0.38	

**Probe pairs**										
**Level**		**Pairs**					**Ratios**			

1		1:3	2:5	4:8			0.33	0.40	0.50	
2		3:7	1:2	3:6	4:6		0.43	0.50	0.50	0.67
3		2:4	3:5	5:7			0.50	0.60	0.71	
4		4:7	2:3	5:6			0.57	0.66	0.83	
5		5:8	3:4	6:7			0.63	0.75	0.86	
6		6:8	4:5	7:8			0.75	0.80	0.88	

**Phase 2**										
**Training pairs**										
**Pairs**	1:8	1:4	9:32	2:6	9:27	8:24	3:8	11:29	12:31	
**Ratios**	0.13	0.25	0.28	0.33	0.33	0.33	0.38	0.38	0.38	

**Probe pairs**										
**Level**		**Pairs**					**Ratios**			

7		10:30	12:30	9:18			0.33	0.40	0.50	
8		12:28	11:22	16:32^†^	14:21		0.43	0.50	0.50^†^	0.67
9		13:26	15:25	17:24			0.50	0.60	0.71	
10		18:32	19:29	15:18			0.56	0.66	0.83	
11		10:16	21:28	19:22			0.63	0.75	0.86	
12		24:32	12:15	22:25			0.75	0.80	0.88	

*Trained pairs were used for rewarded trials in both the training and the test phase. Novel pairs were used only in the probe trials of the test sessions (with each level having its own set of novel pairs). ^†^For all but one of the dogs, the 16:32 pair of numbers (with a ratio of 0.5) was not presented by mistake. Instead, the 16:22 (ratio of 0.73) was used. All wolves were tested with the 16:32 pair.*

**FIGURE 2:**
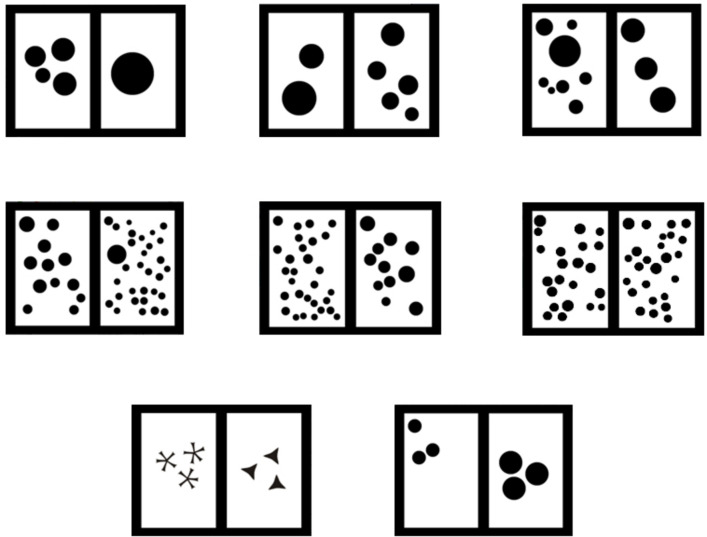
Examples of different stimuli combinations. **(Top row)** “small” numbers: from left to right; 4 vs. 1 (One/Cumulated combination), 2 vs. 5 (Biggest dot/Random combination, largest dot on the left –the lower number), and 8 vs. 3 (Smallest dot/Random, smallest dot on the left –the higher number). **(Middle row)** “large” numbers: from left to right; 12 vs. 31 (Biggest dot/Random, largest dot on the right –the higher number), 29 vs. 11 (Biggest dot/Random, largest dot on the right –the lower number), and 22 vs. 25 (Smallest dot/Random, smallest dot on the right –the lower number). **(Bottom row)** control stimuli: left: combination used for shape control trials, right: combination used for size control trials.

Every stimulus had a constant cumulative surface area (black area) always covering 20% of each side of the screen (thus, cumulative surface area remained constant between the two stimuli of every single pair). Conversely, both dot sizes and positions were randomly chosen with the specification that all dots had a diameter of at least 0.5 cm. Stimuli were presented semi-randomly, with the smaller combination of dots being shown on the left side in half of the trials and on the right side the other half. Some examples of stimulus combinations are provided in Figure 2.

Stevens et al. (2007) proposed that stimulus density (i.e., if the dots are arranged in a clustered or spread manner) could also act as a confounding factor when measuring the ability to discriminate between quantities. Because of this, in trials where the stimulus pair included a single dot (the “one” stimulus), the dots on the other side were clustered (a combination of stimuli we named “One/Cumulated”). Since the position of the dots in all other pairs were pseudo-randomized (in such a manner that none of the dots came in contact with one another), the density between the stimuli was comparable for these other combinations.

Another possible influencing factor would be that the animals made their choice based on simple perceptual rules, such as picking the side with the biggest dot or avoiding the one with the smallest dot. Thus, in half of the trials without a “one,” the side that had the biggest dot was counterbalanced (half of the time on the larger quantity stimulus, half of the time on the smaller quantity stimulus; we named this combination “Biggest dot/Random”) and in the other half of the trials without a “one,” the smallest dot was counterbalanced in the same way (“Smallest dot/Random”).

### General Procedure and Experimental Conditions

#### Ethics Statement

No special permission for use of animals (wolves and dogs) in such socio-cognitive studies is required in Austria (Tierversuchsgesetz 2012–TVG 2012). The relevant committee that allows running research without special permissions regarding animals is: Tierversuchskommission am Bundesministerium für Wissenschaft und Forschung (Austria).

#### General Procedure

In each trial, the subjects were presented with a pair of stimuli, each displayed on each side of the touch screen. These stimuli showed different amounts of dots. Depending on the group they were assigned to, subjects were trained to press the side with the larger amount (group 1) or the side with the smaller one (group 2).

If the subjects made a correct choice, the screen turned white for 1 second, a high-pitched tone would play from the reward dispenser, and a reward would be released. If the choice was incorrect, the screen turned red for 5 s, the computer connected to the touch screen played a lower-pitched tone, and no reward was given. If the animals made an incorrect choice, the trial would be repeated until the subject made the correct choice. The subjects were not restricted, and could freely move toward the touch screen and back at any point during the trials.

Each session consisted of around 30 trials (27–35 depending on the condition and level, see below) plus correction trials. Trials were organized in two phases: phase 1 (in which only “small” numbers were presented) and phase 2 (where “large” numbers were introduced). A training phase would take place before each testing phase. In these training phases, the subjects were trained to discern between different pairs of numbers (shown in Table 1). These “training” pairs from each training phase would then be presented in the subsequent testing phase but they were always rewarded if the choice was correct.

Rewarded trials were interspersed with unannounced “probe” trials to test the numerical competence of the animals. In these probe trials, novel pairs of numbers were presented and no feedback was given (i.e., the screen turned black for 1 s, no tone was played, and no reward was given). The absence of feedback precluded any possibility of learning, since we presented these combinations of quantities more than once. Sessions never started –nor ended– with unrewarded trials; at least 3 rewarded trials were presented in a row before the first unrewarded trial and after the last one.

Each testing phase consisted of 6 levels with 4 sessions each. Levels increased in difficulty by presenting number pairs of increasing ratios in the probe trials (see Table 1).

After the subjects finished either the first or the second testing phase, they were subjected to a control phase, with new stimuli designed to control for any possible non-numerical cues they may have used.

#### Training Phase 1

Training sessions were comprised of 31 trials. In these trials, number pairs with small ratios (up to 0.38) were presented in a randomized sequence (see Table 1), with each pair being shown 3 or 4 times per session.

In four random trials in each session, the outcome was the same as in probe trials (i.e., neither feedback nor a reward was given). This was done to prepare the subjects for the probe trials in test sessions, so that they would be accustomed to getting no feedback for some trials.

The criterion to reach the next phase was to reach at least 80% accuracy at first choice in two consecutive sessions on two different days.

#### Phase 1 (Levels 1–6)

Test sessions were divided in six levels of four sessions each. In these sessions, there were 27 rewarded trials in which the number pairs shown in the training phase were presented (nine different pairs, repeated three times), as well as six (or eight) probe trials; (3 different pairs, repeated twice, 4 in the case of level 2), with new pairs of numbers. In total, each session was composed of 33 trials (or 35 in level 2).

For the dogs, the configuration of the program was changed after starting the experiment. Initially, it was made so that no more than two probe trials were presented in a row. However, when five dogs already performed on the first levels (Toffee: level 4, Ida: level 2, Miley: level 4, Xela: level 4, and Guinness: level 3), we observed that some of them made more mistakes in the trial following a probe trial (usually by pressing again the same side –or promptly changing sides– without noticing the new stimuli), so we discarded all probe trials that took place after another probe trial and excluded them from any analyses. We then changed the configuration so that a probe trial was always followed by a rewarded trial to avoid this effect. All wolves, as well as all other dogs, were tested with the new configuration.

#### Training for Phase 2

This training phase was similar to the first with the exception that no practice probe trials were presented (thus reducing the amount of trials to 27 per session), and that some larger quantities (but still with ratios up to 0.38) were introduced (see Table 1 for details). This time, the criterion to pass to the next phase was at least 85% of correct first choices in two consecutive sessions on two different days. Additionally, it was necessary to make fewer than three wrong first choices on the new larger pairs of stimuli in each of those sessions.

#### Phase 2 (Levels 7–12)

Phase 2 was similar to phase 1 but, once again, some mistakes did take place in the sessions carried out by the wolves. The combinations used in this phase’s probe trials had larger numbers but they were otherwise designed to match as much as possible the ratio of those in the first phase (Table 1).

#### Control Sessions

To further control for the use of non-numerical cues, a final set of six control sessions was run. In these sessions, the rewarded trials were akin to those of the first training phase, but probe trials presented new pairs of stimuli in order to control for the influence of dot size and overall shape of the stimulus. Thus, probe trials in this phase were divided in two types: size control trials and shape control trials. For the size control, we presented the same number of dots on both sides, but they were larger on one side than on the other (in order to check if they used average dot size instead of the number of dots to make their choice; see Figure 2). For the shape control, we showed the subjects another combination of stimuli with the same number and cumulative surface area on both sides but one of the sides had star-like shapes instead of dots, while the other had triangle-like shapes [these two shapes were meant to emulate the appearance of the negative space (white area) between the arrays of large or small amounts of dots; as an asterisk-like form or a triangular-like one, respectively; see Figure 2].

These control sessions were comprised of 27 rewarded trials and six control trials (three for each kind of control), making a total of 33 trials. Overall, every type of control was presented 18 times per subject (21 times in case of some wolves).

### Statistical Analyses

Analyses were conducted with R 3.6.1 (R Core Team, 2019). Performance above chance was tested by using exact binomial tests, comparing the different subsets of the data with a probability of success of 0.5. We also ran generalized mixed models [GLMM; “glmer” function “lme4” package, Bates et al. (2015)] with a binomial distribution and a logit link function, two for each phase of the experiment (one for rewarded trials and one for probe trials). For control trials, models were made for each type of control trial (shape control and size control); and probability of choosing one of the options over the other was analyzed, again, with exact binomial tests. Due to the differences in testing, raising, and housing conditions, all analyses were made separately for dogs and wolves.

The response variable in all models was the first choice of each trial (either “correct” or “wrong”). The rest of the fixed variables and interactions were selected partly based on the Akaike Information Criteria. The “individual” was added as a random effect. Random slopes structure was set with the help of a function devised by Roger Mundry, who also wrote the function we used to check for overdispersion. Models were analyzed with Wald χ^2^ tests (“car” package; Fox and Weisberg, 2018) to detect effects.

As the stimulus that was presented in the correct side was a nested variable of the pair of stimuli presented, *post hoc* Wilcoxon tests were used to compare the probability of success for both of the stimuli within each pair (adjusting the *p*-value according to the Holm method for multiple comparisons).

Due to the results of control sessions, an additional *post hoc* model was run to assess the potential use of non-numerical cues by some of the dogs (more details in the results section). Graphs were created through the ggplot2 (Wickham, 2016), sjPlot (Lüdecke, 2018b), and sjmisc (Lüdecke, 2018a) packages.

## Study 1: Dogs

### Subjects

Thirty-one pet dogs aged 1–9 years were recruited from an existing pool of subjects and their owners at the Clever Dog Lab, Vienna. Eleven of these dogs (two of them with previous touch screen experience and nine of them without it) dropped out during the pre-training phase due to lack of motivation, leaving a final sample size of *n* = 20, with an average age of 4.51 ± 2.62 years. Dogs were assigned semi-randomly to the two experimental groups. Sex (6–8 females and 3 males per group), and age (on average 4.60 ± 2.86 years for group 1 and 4.43 ± 2.41 for group 2) were counterbalanced as much as possible. Dogs from various breeds were tested (see Supplementary Table 1 in the Supplementary Material for further details) and were as well matched to the extent possible between the groups.

### Testing Facility and Set-Up

The study took place in a 2.9 m × 3.5 m room of the Clever Dog Lab in Vienna. At the first appointment the dogs had time to familiarize themselves with the room and the experimenter, to feel comfortable in the situation. Training and testing sessions took place once or twice per week for about 30–60 min each. For shaping and rewarding, positive reinforcement was used exclusively (further details about the pre-training are provided in the Supplementary Material).

The touch screen used for the dogs had a resolution of 1024 pixels × 748 pixels, and the glass panes in front of it were 13.5 cm × 22 cm each. The distance between screen and dispenser was 2.5–2.8 m, to ensure the animals moved away from the screen and therefore observed the new stimuli before making a choice in the next trial. For three dogs (Bertl, Chilly, and Flora) the distance was reduced by half due to them approaching the dispenser slower than the rest of the subjects. To avoid any unintentional cues given by the owner or the experimenter, a plywood panel was fixed next to the screen. Both the owner and experimenter remained behind this panel from the first training phase onwards.

### Results

#### Training Phase 1

Out of the 20 dogs trained, two of them (Shiloh and Flora) did not achieve the testing criterion and dropped out of the experiment. Another two dogs (Chilly and Havanna) completed this training phase but did not continue with the rest of the experiment. The remaining 16 dogs continued with the phase 1 of the study.

The dogs that completed the training session required a varying amount of trials to achieve the learning criterion, averaging at 626.47 ± 335.58.

#### Test Phase 1

##### Training trials

The performance on the trials after a probe trial varied significantly from the other rewarded trials (GLMM: z = 6.110, *p* < 0.001). Thus, these trials were excluded from further analyses.

Performance was above chance on the remaining rewarded trials (binomial test: probability of success = 0.908, C.I. 95%: 0.901–0.914, *p* < 0.001). Furthermore, dogs chose the correct side above chance for every single combination of numbers (see Table 2). Probability of success was also above chance for every combination of stimuli (binomial test; biggest circle/random: probability of success = 0.829, C.I. 95%: 0.811–0.846, *p* < 0.001; smallest circle/random: probability of success = 0.903, C.I. 95%: 0.889–0.917, *p* < 0.001; one/cumulated: probability of success = 0.941, C.I. 95%: 0.934–0.948, *p* < 0.001).

**TABLE 2:** Probabilities of success for the pairs of numbers presented in rewarded trials in the first phase of study 1 (dogs).

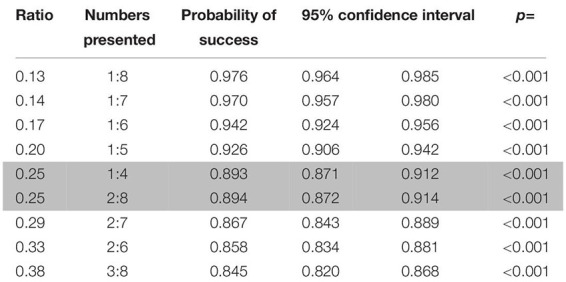

*Probability of success calculated with exact binomial tests, comparing performances with a 0.5 probability of success by chance. Rows with the same background color indicate number pairs with the same ratio.*

###### Factors affecting the performance of the animals

**FIGURE 3:**
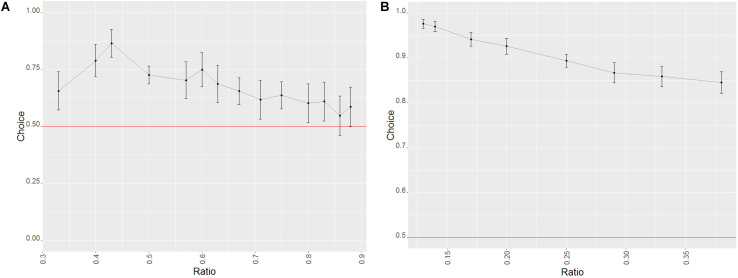
Effect of the ratio on the probability of success in trials of phase 1 of study 1 (dogs). Error bars set at the 95% confidence interval; the red line represents probability of success by chance. **(A)** Probe trials. **(B)** Training trials.

Despite the fact that the animals performed above chance on every number pair (Table 2) as well as each of the combinations used to control for perceptual features, success was dependent on ratio (decreasing as the ratio increased, see Figure 3; Wald χ^2^ = 39.860, *p* < 0.001) and the combination of stimuli (Wald χ^2^ = 29.769, *p* < 0.001). The combination that controlled for the side with the biggest dot yielded the lowest amount of correct choices (biggest dot/random vs. smallest dot/random: *z* = 2.167, *p* = 0.030; biggest dot/random vs. one/cumulated: *z* = 4.680, *p* < 0.001). When further analyzing performance within each combination of stimuli, we only found significant differences in the biggest dot/random trials, with fewer correct choices when the stimulus with the biggest dot was not the correct one (Wilcoxon: *W* = 428744, *p* < 0.001). We found no such differences in the other combination of stimuli (Wilcoxon: smallest dot/random: *W* = 392486, *p* = 1; one/cumulated: *W* = 2424752, *p* = 1). This suggests that dogs used the size of the dots presented to make their choices, picking the side with the largest dot significantly more than the other stimulus.

We also found a learning effect (Wald χ^2^ = 16.5484, *p* = 0.005), with the performance of every level aside for the second being significantly better than the first one (GLMM: level 1 vs. 2: *z* = 1.208, *p* = 0.227; level 1 vs. 3: *z* = 1.985, *p* = 0.047; level 1 vs. 4: *z* = 2.292, *p* = 0.022; level 1 vs. 5: *z* = 3.489, *p* < 0.001; level 1 vs. 6:*z* = 2.854, *p* = 0.004). An effect of group was also found, but there were no significant differences between the groups (Wald χ^2^ = 4.780, *p* = 0.029; GLMM: group 1 vs. group 2 *z* = −1.413, *p* = 0.157).

##### Test (probe) trials

Dogs performed above chance on probe trials (binomial test: probability of success = 0.677, C.I. 95%: 0.657–0.696, *p* < 0.001). Performance was above chance for all number pairs except for 6:7 and 7:8 (see Table 3) and in all combinations of stimuli (binomial test; biggest dot/random: probability of success = 0.662, C.I. 95%: 0.632–0.690, *p* < 0.001; smallest dot/random: probability of success = 0.702, C.I. 95%: 0.673–0.729, *p* < 0.001; one/cumulated: probability of success = 0.638, C.I. 95%: 0.575–0.698, *p* < 0.001).

**TABLE 3:** Probabilities of success for the different number pairs presented in probe trials in the first phase of study 1 (dogs).

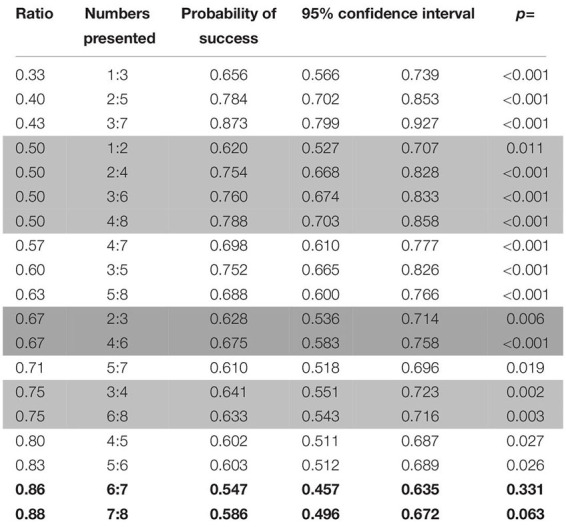

*Probability of success calculated with exact binomial tests, again a 0.5 probability of success by chance. Number pairs with the same ratio are shown with the same background color. Rows with numbers in bold indicate that success was not above chance level for that pair of numbers.*

###### Factors affecting the performance of the animals

Success in probe trials was influenced by the ratio (with higher ratios yielding worse performances, see Figure 3; Wald χ^2^ = 47.334, *p* < 0.001) as well as the combination of stimuli presented (Wald χ^2^ = 14.552, *p* < 0.001), although no difference between the three combinations was found (GLMM: biggest dot/random vs. one/cumulated: *z* = −1.212, *p* = 0.226; biggest dot/random vs. smallest dot/random: *z* = 1.028, *p* = 0.304). When taking into consideration only biggest dot/random trials, dogs were more successful when the side with the biggest dot was the correct one (Wilcoxon: *W* = 151528, *p* = 0.003), which again suggested that dogs used the size of the dots at least to some degree to make their decisions. We found no such differences in any of the other combinations of stimuli (Wilcoxon: smallest dot/random: *W* = 140156, *p* = 1; one/cumulated: *W* = 8258, *p* = 0.259).

#### Training Phase 2

Only eight dogs out of the 16 that completed the previous test phase were trained for the second phase of the study (although all of them did participate in the control phase later on). Three of those subjects (Ida, Oszkar, and Miley) did not complete this training phase, leaving a total of five dogs that continued toward phase 2 of the experiment. The dogs that completed the second training phase required an average of 873.00 ± 690.91 trials to do so.

#### Phase 2

##### Training trials

As was the case in Phase 1, there was a significant variation in performance on trials after a probe trial (GLMM: *z* = 4.360, *p* < 0.001). Therefore, those trials were excluded from any further analyses.

Dogs correctly chose the respective stimuli above chance in the rewarded trials of the second phase (binomial test: probability of success = 0.849, C.I. 95%: 0.834–0.863, *p* < 0.001), and the same applied to every pair of numbers (see Table 4). Performance was also above chance for every combination of stimuli (binomial test; biggest circle/random: probability of success = 0.806, C.I. 95%: 0.779–0.831, *p* < 0.001; smallest circle/random: probability of success = 0.889, C.I. 95%: 0.867–0.908, *p* < 0.001; random/cumulated: probability of success = 0.852, C.I. 95%: 0.820–0.881, *p* < 0.001).

**TABLE 4:** Probabilities of success for the ratios and number pairs presented in rewarded trials in the second phase of study 1 (dogs).

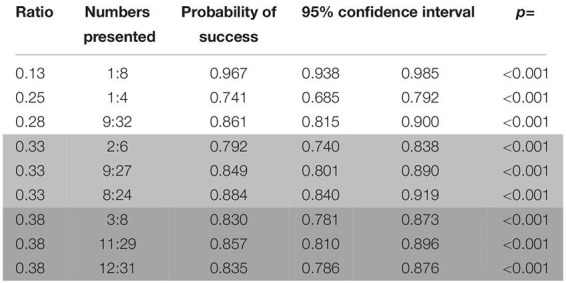

*Probability of success calculated with exact binomial tests, comparing performances with a 0.5 probability of success by chance. Rows with the same background color indicate pairs of numbers with the same ratio.*

###### Factors affecting the performance of the animals

**FIGURE 4:**
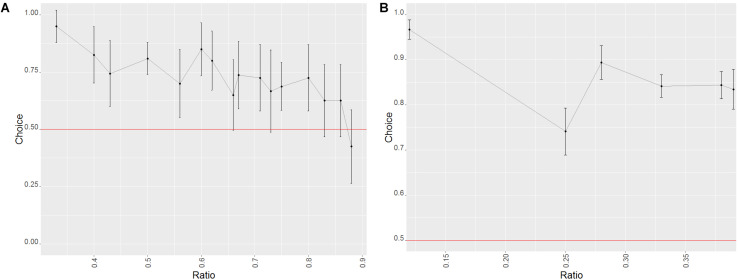
Effect of the ratio on the probability of success in the trials of phase 2 of study 1 (dogs). Error bars set at the 95% confidence interval; the red line represents probability of success by chance. **(A)** Probe trials. **(B)** Training trials.

As was the case for the rewarded trials of phase 1, success decreased as the ratio increased (Wald χ^2^ = 21.681, *p* < 0.001; see Figure 4) and the combination of stimuli influenced performance with the combination biggest dot/random yielding worse performances than both smallest dot/random, and one/cumulated (Wald χ^2^ = 24.093, *p* < 0.001; GLMM: *z* = 1.981, *p* = 0.048; *z* = 3.797, *p* < 0.001). Within the different combinations of stimuli, once again, we found differences only in the combination that controlled for side of the biggest dot, with worse performances when the side with the largest dot wasn’t the correct one (Wilcoxon: *W* = 121173, *p* < 0.001). No differences were found in the other stimulus combinations (Wilcoxon: small dot/random: *W* = 123683, *p* = 1; one/cumulated: *W* = 36949, *p* = 1).

##### Test (probe) trials

Overall probe trials in the second phase were performed above chance level (binomial test: probability of success = 0.730, C.I. 95%: 0.697–0.762, *p* < 0.001). All pairs of numbers representing a ratio below 0.63 were selected above chance. Above that, only 14:21, 17:24, 21:28, and 12:15 were successfully discriminated (see Table 5). Probability of success remained above chance for both combinations of stimuli (binomial test; biggest dot/random: probability of success = 0.695, C.I. 95%: 0.646–0.741, *p* < 0.001; smallest dot/random: probability of success = 0.765, C.I. 95%: 0.720–0.808, *p* < 0.001; since none of the number pairs contained a “one” stimulus, the “one/cumulated” combination was not present in these trials).

**TABLE 5:** Probabilities of success for the different ratios and pairs of numbers presented in probe trials in the second phase of study 1 (dogs).

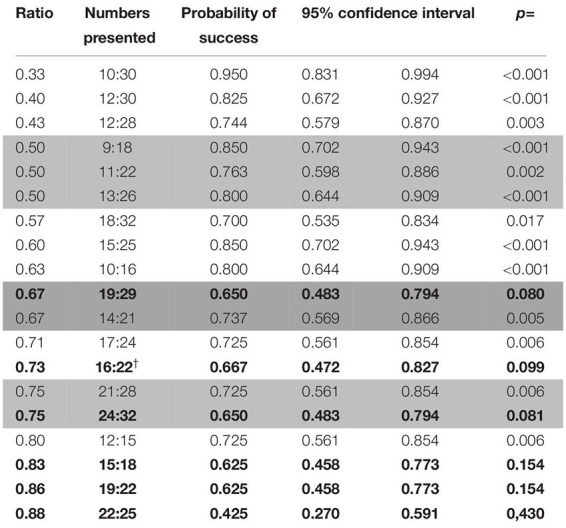

*Probability of success calculated by using exact binomial tests, comparing the subjects’ performances with a 0.5 probability of success by chance. Pairs of numbers with the same ratio share a background color in the table. Numbers in bold indicate that the probability of success for that number pair was not above chance. ^†^The 16:22 pair of numbers was mistakenly presented instead of the intended one (16:32) for all but one of the dogs. As only one of the subjects was tested for the correct pair, we didn’t analyze its probability of success.*

###### Factors affecting the performance of the animals

For the test trials in this phase, only the ratio (Wald χ^2^ = 7.568, *p* = 0.006) had a significant effect (with a decrease in success the higher the ratio; see Figure 4), as the effect of the stimulus combination was non-significant (Wald χ^2^ = 2.721, *p* = 0.099). This seems to imply that the dogs’ overall bias toward the side with the largest dot was absent in probe trials for the second phase.

#### Control Phase

All dogs that completed at least one of the test phases –except for Oszkar and Guinness– participated later on the control phase, leaving a sample size of 14 for this phase of the study.

##### Shape control

Dogs chose the side with triangular shapes over the one with stars (binomial test: probability of choosing triangles = 0.676, C.I. 95%: 0.613–0.735, *p* < 0.001). Furthermore, the individuals from group 2 (the ones trained to choose the smaller number of dots) chose triangles significantly more than the other group (GLMM: *z* = 4.265; *p* < 0.001). However, individuals from group 1 did not show this preference for the triangular shape (binomial test: probability of choosing triangles = 0.484, C.I. 95%: 0.394–0.575, *p* = 0.789).

##### Size control

Our subjects chose the side with the largest dots over the one with the smallest ones (binomial test: probability of choosing bigger dots = 0.770, C.I. 95%: 0.711–0.822, *p* < 0.001). As with the shape control, the group that was trained for fewer dots chose larger dots significantly more than the other group (GLMM: *z* = 4.525, *p* < 0.001). However, in this case, the group trained for the bigger amount did also choose the larger dots above chance (binomial test: probability of choosing bigger dots = 0.627, C.I. 95%: 0.536–0.711, *p* = 0.006).

#### *Post hoc* Average Size Control

Due to the results of the control phase and the detailed analyses of the various experimental phases, we ran a final model to assess the performance of both groups in only the trials with the number combinations with the most similar inter-stimulus dot size: the pairs differing in just one number (i.e., 1:2, 2:3, 3:4, 4:5, 5:6, 6:7, and 7:8).

There was no significant effect of group in these trials, nor of the interaction between group and the pair of stimuli (Wald χ^2^ = 0.077, *p* = 0.781; Wald χ^2^ = 0.054, *p* = 0.973; respectively). As stated above, dogs performed above chance level in all number pairs except for 6:7 and 7:8.

However, we also did not find any effect from any of the variables used in other models (including ratio: Wald χ^2^ = 2.498, *p* = 0.114; see Supplementary Figure 1).

### Discussion for Study 1

Our data suggest that pet dogs are able to distinguish between two numbers, and that their performance in these quantity discrimination tasks decreases as the ratio increases (as predicted by Weber’s law). Nonetheless, continuous variables (such as dot size) seem to have influenced the performance of our subjects throughout the study. Contrary to both the results of previous studies and our own predictions, dogs were able to distinguish ratios higher than 0.50. More than that, in phase 1, they succeeded in almost every single combination for which they had received no training and they still had a good rate of success on the combinations with higher magnitudes in phase 2.

There are several possibilities that may explain this higher level of performance. One of them is that the use of the touch screen removes the confounding effect of the presence of food. In other studies, food was given to the dogs whether or not they made the correct choice, which made even wrong choices not very costly. Fernand et al. (2018) found a similar effect in a reverse-reward contingency task, in which the dogs kept choosing the larger stimulus, even though the outcome of that choice would leave them with a smaller reward.

Also to be taken into consideration is the relative difficulty of this task when compared to the sequential procedure we previously used (see Range et al., 2014). The sequential paradigm required the subjects to keep in mind both quantities before drawing comparisons, a process that may have been too cognitively demanding. Conversely, the subjects were able to perceive both stimuli at the same time in this study, which may have facilitated their choices, thus improving their performance. Nonetheless, Miletto Petrazzini and Wynne (2017) presented both stimuli simultaneously and their results seem to match those of the sequential task, so other factors may be at play as well such as the extensive training phase.

Previous studies focused on spontaneous choices (toward the highest quantity of food items), while we exposed our subjects to extensive training to induce them to choose either the highest or lowest amount of items. Some authors have drawn attention to the possibility that giving animals extensive training to perform a quantity discrimination task may re-purpose neuro-cognitive systems that are normally not concerned with numerical competence, so it is certainly possible that our training may have not only taught the subjects “which stimulus to choose” but also “how to choose better in general” (Barnard et al., 2013; see Agrillo and Bisazza, 2014 for a detailed comparison between the spontaneous and trained approaches). In guppies, for example, extensive training has been shown to increase numerical competence skills (Bisazza et al., 2014). Accordingly, our extensive training might have directed their attention to the relevant features of the task (quantity) making it easier to successfully choose between the harder pairs of stimuli that they were not trained for (Zentall and Riley, 2000). Further research with both spontaneous choice and training procedure paradigms should be done to further explore the mechanisms behind this difference in performance.

In any case, the procedure used seems to have an influence on the outcome of quantity discrimination tasks in dogs. This is not an isolated case, as angelfish show a higher upper limit of ratio when tested to choose the highest amount of food than when approaching the biggest shoal (Gómez-Laplaza and Gerlai, 2011; Gómez-Laplaza et al., 2018). Just as Gómez-Laplaza et al. (2018) discussed, differences in motivation (in this case, only receiving a reward with correct choices) and cognitive abilities required by the task (perceiving both numbers at once instead of having to remember them) may have driven the contrast between our results and those found previously in the literature.

## Study 2: Wolves

### Subjects

Eleven wolves participated on this study, with ages averaging 3.77 ± 1.08 years. All of the wolves had previous touch-screen experience (see Supplementary Table 2 in the Supplementary Material for further details).

All of the wolves were hand-raised with conspecifics in peer groups, after being separated from their mothers in the first 10 days after birth. They were bottle-fed and later hand-fed by humans and had continuous human interaction in the first 5 months of their lives. After that, they were introduced into packs with other adult wolves and currently live in large 2,000–8,000 m^2^ enclosures.

The wolves were tested in a 2.6 m × 3 m room at the Wolf Science Center in Ernstbrunn, Austria. All subjects were familiarized with the room prior to the sessions (usually ranging between 10 and 45 min) and were, overall, conducted with less regularity compared to the dogs; sessions usually took place only once a week except for breeding season (when some animals would refuse to work) or when other tests were carried out. A maximum of one session per day was carried out for every subject.

As was the case for the dogs, the wolves were divided into two groups (with group 1 being trained to choose big numbers, and group 2 to choose small numbers). Further, like the dogs, we counterbalanced the groups by sex (3 males and 2 or 3 females per group) and age (3.96 ± 0.67 years for group 1 and 3.61 ± 1.32 years for group 2).

Whenever testing was interrupted for a long period of time, the subjects would have to repeat the respective training phase until they achieved once again the learning criterion.

### Testing Facility and Set-Up

The touch screen used for the wolves was similar to that used for the dogs, with a few differences in size (the glass panes having a size of 20 cm × 26 cm, and the resolution of the screen being 1,920 pixels × 1,080 pixels). Since the treat dispenser did not work well with the food rewards used for the wolves (mixture of dry food and meat), the experimenter would reward the subjects when their choices were correct and, instead of the dispenser playing a sound, the experimenter would use the sound of a clicker as a reinforcer. To avoid any possible experimenter cues, the experimenter stood on the side opposite to the touch screen, next to the dispenser (which made it impossible for the subjects to look at the experimenter at the same time they were making a choice), and was otherwise instructed to maintain a neutral facial expression during the trials. The distance between the treat dispenser and the touch screen was the same as that used for the dogs. No plywood panel was placed next to the screen for this experiment.

Stimuli and procedures for the wolves were identical as the ones used for the dogs.

### Results

#### Training for Phase 1

Two of the wolves that were trained for this task (Geronimo and Yukon) did not achieve the testing criterion and were subsequently excluded from the experiment. The remaining nine wolves completed the training phase in an average of 486.86 ± 426.77 trials. Due to problems during the data management process, the records for the training trials of two of the subjects (Aragorn and Shima) were lost, and thus are not included in this analysis.

#### Phase 1

Some of the sessions in this phase (the last two sessions of level 4 for Una, the first two sessions of level 5 for Aragorn, and the first two sessions of level 4 as well as the entirety of level 3 for Amarok) were lost during the data management process, and thus are not included in any of the analyses. Furthermore, due to human error, some individuals received more than four sessions per level, but these additional sessions were not included in any analysis either.

Eight of the nine individuals tested in this phase completed all 6 levels; Una was eventually dropped (after she completed level 5) due to lack of motivation.

##### Training trials

Performance on trials after a probe trial differed significantly from the rest of the rewarded trials (GLMM: *z* = 4.192, *p* < 0.001), and as such, they were excluded.

Performance was above chance on training trials (binomial test: probability of success = 0.873, C.I. 95%: 0.863–0.883, *p* < 0.001), and every number combination used was correctly selected above chance level as well (see Table 6). Probability of success was also above chance for all three combinations of stimuli (binomial test; biggest circle/random: probability of success = 0.757, C.I. 95%: 0.728–0.784, *p* < 0.001; smallest circle/random: probability of success = 0.886, C.I. 95%: 0.864–0.906, *p* < 0.001; one/cumulated: probability of success = 0.916, C.I. 95%: 0.903–0.927, *p* < 0.001).

**TABLE 6:** Probabilities of success for the pairs of numbers presented in rewarded trials in the first phase of study 2 (wolves).

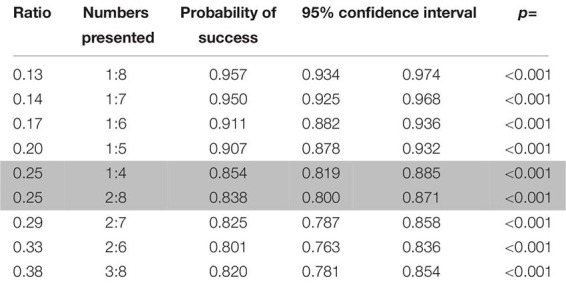

*Probability of success calculated with exact binomial tests, comparing performances with a 0.5 probability of success by chance. Rows with the same background color indicate number pairs with the same ratio.*

###### Factors affecting the performance of the animals

**FIGURE 5:**
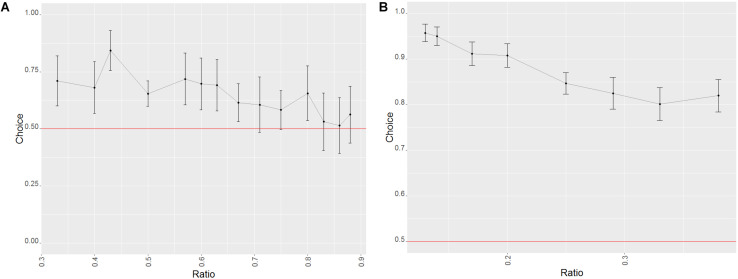
Effect of the ratio on the probability of success in the trials of phase 1 of study 2 (wolves). Error bars set at the 95% confidence interval; the red line represents probability of success by chance. **(A)** Probe trials. **(B)** Training trials.

Even though all number pairs were correctly chosen above chance level, success was still affected by ratio (with a lower amount of successful choices the higher the ratio; Wald χ^2^ = 14.566, *p* < 0.001, see Figure 5) and combination of stimuli (with the biggest dot/random combination yielding significantly worse results than the one/cumulated combination: Wald χ^2^ = 36.887, *p* < 0.001; GLMM: *z* = 0.756, *p* < 0.001). Furthermore, we did find a significant difference in performance within the biggest dot/random trials, with overall fewer correct choices when the side with the largest dot was not the correct one (Wilcoxon: *W* = 428744, *p* < 0.001), which does suggest that our subjects may have been using this stimulus as a non-numerical cue to inform their choices. No differences were found between the stimuli of the smallest dot/random and one/cumulated combinations (Wilcoxon: *W* = 105088, *p* = 1, Wilcoxon: *W* = 676750, *p* = 1; respectively).

##### Test (probe) trials

Performance on unrewarded probe trials was above chance level (binomial test: probability of success = 0.644, C.I. 95%: 0.618–0.671, *p* < 0.001), and the same remained true for every combination of stimuli (binomial test; biggest circle/random: probability of success = 0.619, C.I. 95%: 0.576–0.660, *p* < 0.001; smallest circle/random: probability of success = 0.658, C.I. 95%: 0.618–0.696, *p* < 0.001; one/cumulated: probability of success = 0.685, C.I. 95%: 0.602–0.760, *p* < 0.001). Most number pairs up to a ratio of 0.80 were successfully discriminated by the subjects (except for 2:3, 5:7; and 6:8; see Table 7).

**TABLE 7:** Probabilities of success for the different number pairs presented in probe trials in the first phase of study 2 (wolves).

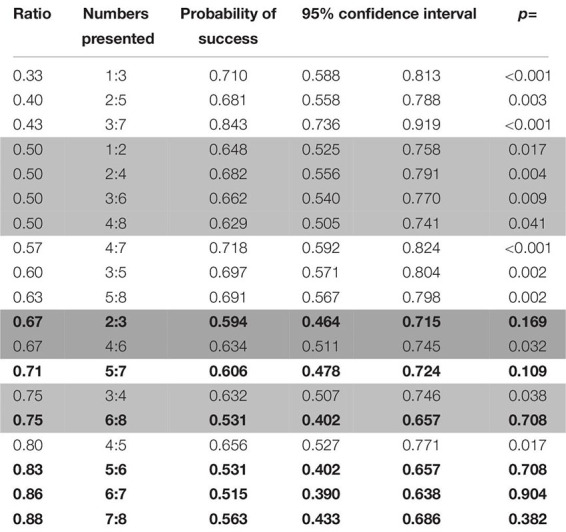

*Probability of success calculated with exact binomial tests, again a 0.5 probability of success by chance. Number pairs with the same ratio are shown with the same background color. Rows with numbers in bold indicate that success was not above chance level for that pair of numbers.*

###### Factors affecting the performance of the animals

Only the ratio had an effect in probe trials (Wald χ^2^ = 10.038, *p* = 0.002; see Figure 5), with no perceived differences between the different combination of stimuli (Wald χ^2^ = 1.574, *p* = 0.455), suggesting a reduced use of non-numerical information in probe trials when compared to the number pairs that the subjects were trained for.

#### Training for Phase 2

Six out of the eight wolves that completed phase one went through the training for phase 2. The average number of trials they needed to proceed to the test phase was of 2702.00 ± 2092.97.

#### Phase 2

A sizable amount of the records for the sessions in this phase were lost due to complications in the data management process (the entirety of levels 2, 3, 4, and 5 for Chitto, as well as the two last sessions of level 1 and the first of level 6; levels 5 and 6 for both Nanuk and Shima; level 2 and the first session of level 3 for Aragorn; and the last session of level 4 for Kaspar). These sessions were thus not included in any of the analyses.

Furthermore, as with the first phase of the experiment, some individuals received additional sessions after their fourth for some of the levels due to human error. Once more, these trials were excluded from analyses.

##### Training trials

We found the performance of rewarded trials to be significantly different when the trials after a probe trial were taken into consideration (GLMM: *z* = 4.192, *p* < 0.001), so those trials were excluded from any further analyses.

Probability of success remained above chance level for the training trials of this phase (binomial test: probability of success = 0.829, C.I. 95%: 0.812–0.844, *p* < 0.001), and so did the probability of success for every combination of stimuli (binomial test; biggest circle/random: probability of success = 0.752, C.I. 95%: 0.720–0.781, *p* < 0.001; smallest circle/random: probability of success = 0.854, C.I. 95%: 0.828–0.876, *p* < 0.001; one/cumulated: probability of success = 0.913, C.I. 95%: 0.884–0.936, *p* < 0.001). All number pairs were successfully discerned above chance levels (see Table 8).

**TABLE 8:** Probabilities of success for the pairs of numbers presented in rewarded trials in the second phase of study 2 (wolves).

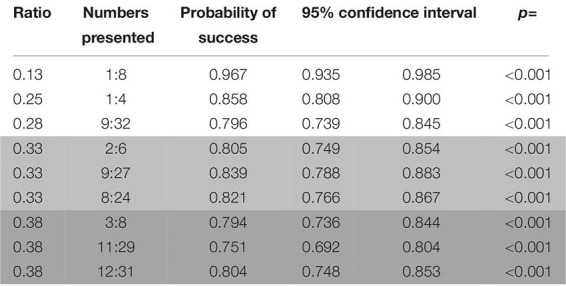

*Probability of success calculated with exact binomial tests, comparing performances with a 0.5 probability of success by chance. Rows with the same background color indicate number pairs with the same ratio.*

###### Factors affecting the performance of the animals

**FIGURE 6:**
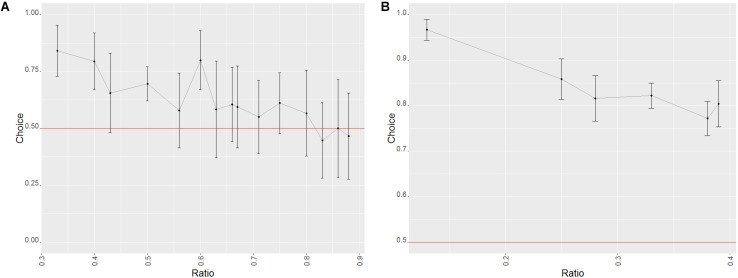
Effect of the ratio on the probability of success in the trials of phase 2 of study 2 (wolves). Error bars set at the 95% confidence interval; the red line represents probability of success by chance. **(A)** Probe trials. **(B)** Training trials.

Similarly to the training trials in phase 1, we found an effect both of the ratio (Wald χ^2^ = 14.066, *p* < 0.001; see Figure 6) and the combination of stimuli, with a significant difference between the biggest dot/random and the one/cumulated combinations (Wald χ^2^ = 17.443, *p* < 0.001; GLMM: *z* = 2.148, *p* = 0.032) on this type of trials in phase 2. We also found a decrease in successful trials whenever the side with the biggest dot was not the correct one within the biggest dot/random combination of stimuli (Wilcoxon: *W* = 87546, *p* = 0.023), indicating, once again, a possible use of the side with the largest dot as a non-numerical cue to solve the task. No such effects were found for the other combinations of stimuli (smallest dot/random: Wilcoxon: *W* = 93489, *p* = 1; one/cumulated: Wilcoxon: *W* = 28251, *p* = 1).

##### Test (probe) trials

Success in probe trials was overall above chance level (binomial test: probability of success = 0.642, C.I. 95%: 0.604–0.679, *p* < 0.001), which was also the case for both combinations of stimuli (binomial test; biggest dot/random: probability of success = 0.634, C.I. 95%: 0.582–0.684, *p* < 0.001; smallest dot/random: probability of success = 0.651, C.I. 95%: 0.594–0.705, *p* < 0.001; the “one/cumulated” combination was not present in these trials). Regardless, only six number pairs (10:30, 12:30, 9:18, 13:26, 15:25, and 19:29) were discriminated above chance levels (see Table 9).

**TABLE 9:** Probabilities of success for the different ratios and pairs of numbers presented in probe trials in the second phase of study 2 (wolves).

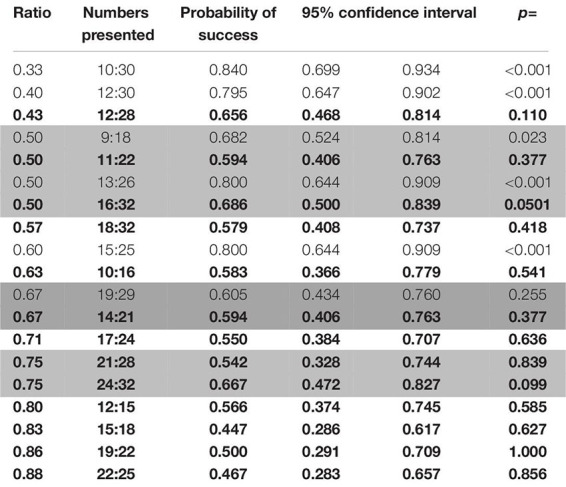

*Probability of success calculated by using exact binomial tests, comparing the subjects’ performances with a 0.5 probability of success by chance. Pairs of numbers with the same ratio share a background color in the table. Numbers in bold indicate that the probability of success for that number pair was not above chance.*

###### Factors affecting the performance of the animals

As with the probe trials in phase 1, ratio was the only factor that had any bearing on performance (Wald χ^2^ = 22.474, *p* < 0.001; see Figure 6), as we found no effect of the combination of stimuli (Wald χ^2^ = 0.286, *p* = 0.5932).

#### Control Phase

All of the wolves that participated on the second phase of the experiment were also subjected to the control sessions. However, the records from one of these wolves (Shima) were lost due to problems in data management, and thus not included for any of the analyses.

##### Shape control

We did find an overall preference toward the “triangle” shape for individuals of both groups (binomial test: probability of choosing triangle = 0.822, C.I. 95%: 0.727–0.895, *p* < 0.001). Furthermore, individuals from group 2 (trained to choose the smaller amount) chose this shape significantly more often than subjects from group 1 (GLMM: *z* = 2.963, *p* = 0.003).

After further inspection, we found that the individuals from group 2 chose the triangular shape above chance levels (binomial test: probability of choosing triangle = 0.926, C.I. 95%: 0.821–0.979, *p* < 0.001), but those in group 1 did not (binomial test: probability of choosing triangle = 0.667, C.I. 95%: 0.490–0.814, *p* = 0.065).

##### Size control

When subjects from both groups were taken into account, we did not find any preference toward any of the sizes of the dots (binomial test: probability of choosing the larger dots = 0.444, C.I. 95%: 0.340–0.553, *p* = 0.343). However, we did find an effect of group in our model, with subjects from group 2 showing a preference for the larger dots (GLMM: *z* = 1.997, *p* = 0.046).

After further examination, we observed that the individuals from group 1 chose the stimulus with the smaller dots above chance (binomial test: probability of choosing the larger dots = 0.306, C.I. 95%: 0.163–0.481, *p* = 0.029) and that group 2 has done the same with the stimulus with the larger dots (binomial test: probability of choosing the larger dots = 0.722, C.I. 95%: 0.585–0.835, *p* = 0.001).

#### *Post hoc* Average Size Control

To test the implications of the results of the control trials on the wolves’ use of non-numerical cues, we once again ran another model with the data from the combinations differing only in one number (as they would have the least differences in overall dot size between stimuli).

We found an effect from the interaction between ratio and group, with the individuals from group 1 showing better performances in trials with higher ratios, as opposed to group 2 (Wald χ^2^ = 8.067, *p* = 0.005; see Supplementary Figure 2 in the Supplementary Material). No further effects were found (including ratio: Wald χ^2^ = 0.531, *p* = 0.466; see Supplementary Figure 3 in the Supplementary Material).

Out of the number pairs used in this model, some of them (1:2, 3:4, and 4:5) were performed above chance, while the others (2:3, 5:6, and 7:8) were not, as stated above (see Table 7).

### Discussion for Study 2

In line with the available literature, we found that wolves are able to distinguish between quantities of increasing ratios. Both Utrata et al. (2012) and Miletto Petrazzini and Wynne (2017) showed that wolves are able to discern between quantities differing in ratios up to 0.75, comparable to the maximum of 0.80 that our subjects were able to distinguish above chance in the current study.

We did find, however, an effect of ratio on our subjects’ performance with worse performance at higher ratios, in accordance with Weber’s law, something that these previous studies did not show. This suggests that the limited numbers used on those paradigms (a maximum of four items to count) may not have been enough to find any difference in performance for different ratios (due to either a ceiling effect or the lack of enough pairs of numbers to find any pattern from their performances). However, it could tentatively provide support as well for the object-file system, which postulates that “low” quantities are processed in a faster, more accurate fashion than “higher” ones (usually up to a maximum of four; Feigenson et al., 2004; Agrillo et al., 2012). Nevertheless, our wolves were not able to distinguish between the stimuli in the 2:3 pair, which would go against this system’s predictions as both numbers are low, and yet they were unable to differentiate them above chance.

As expected, the overall results in phase 2 (where higher numbers were used) were considerably worse than those of phase 1 (as fewer number pairs were chosen correctly above chance levels, even though their ratios were comparable to those presented during phase 1). However, due to the very limited data availability for this part of the experiment, we cannot draw any conclusions from this sample.

Finally, we need to draw attention to the fact that several subjects carried out additional sessions in some of the levels due to human error (see Supplementary Table 3 for more details). Although unlikely, given that the number pairs used in probe trials were exclusive to each level, it remains a possibility that the additional trials may have somehow affected the wolves’ performance in the test trials. In contrast, training trials could certainly have been affected by this. However, since we did not find any effect of the level on the probability of success on training trials –in other words, no learning effect– the additional sessions are unlikely to have affected the performance.

## General Discussion

As expected, we found an effect of ratio for both of our species, in accordance with Weber’s Law. Dogs were able to discern combinations they were not trained for with ratios up to 0.83 in phase 1, and up to 0.80 in phase 2 (although performance did decrease after 0.63; some combinations above this ratio were discerned correctly above chance while others were not). Wolves, on the other hand, correctly distinguished number pairs with a ratio of up to 0.80 in phase 1, and up to 0.60 in phase 2 (with some pairs with lower ratios not being correctly distinguished in both phases). The seemingly worse results in phase 2 for both species seem to imply that there may also be an effect of magnitude at play, which we had predicted.

We did not study, however, the possibility that a separation between two distinct number processing systems (the object-file system and the analog magnitude system) does take place in canids. According to some authors, the discrimination of quantities up to four is regulated by the object-file system (Feigenson et al., 2004; Agrillo et al., 2012), and those above that number are the domain of the analog magnitude system. In the current study, however, we aimed at testing the subject species at overall high magnitudes, so we decided to make the split between the “high” and “low” numbers at eight, as it provided us with more possible number pairs with the same ratios between both phases of the experiment. Future studies should focus on looking for evidence of presence or absence of both of these systems in dogs and wolves, as well as finding the upper limit for the object-file system if its presence is indeed confirmed.

It remains unclear why some of the combinations were not distinguished when some others with higher ratios were, especially in phase 2 for both of our species. Since this effect seems to be more pronounced in wolves (both being the species with the least amount of individuals tested and the one with a noticeably incomplete dataset), and these fluctuations being present only in phase 2 for dogs (with a drastically reduced amount of individuals), we presume this outcome to be a consequence of the reduced sample size. Still, this difference may have also been partly influenced by the combined effect of ratio and magnitude, with both species failing to distinguish above chance some of the pairs with the highest magnitudes (e.g., 19:29 and 24:32). Future studies should focus on the performance of these species when distinguishing pairs of high magnitudes.

It is worth noting that wolves seem to have performed worse than dogs overall, with fewer number pairs correctly chosen above chance, which could indicate that the wolves were not as focused on the task as the dogs. Wolves’ sessions were generally shorter than dogs’ (ranging from 10 to 45 min in wolves and 30–60 in dogs), so it is possible that they made their decisions faster, which could have led to an overall higher amount of incorrect choices. Additionally, it is likely that the pet dogs were more at ease within the testing room due to their different upbringing (as they would generally spend more time indoors than the wolves), which could have negatively impacted their concentration.

It is also possible that the less frequent sessions when compared with the dogs (with some individuals participating in this experiment for several years, see Supplementary Table 2 for more details) may have also negatively influenced the wolves’ learning of the skill. Indeed, wolves needed on average 2702.00 ± 2092.97 trials to achieve learning criterion for the second phase of the study, while dogs did it in 873.00 ± 690.91 trials. In general, however, it is important to keep in mind that the wolves and dogs tested here were not comparable in terms of life and experimental experiences, which is why we refrain from drawing direct comparisons here and would also like to caution the against arriving at unwarranted conclusions.

It is up for debate, however, how much of these differences in performance may have come as an actual variation of cognitive abilities and not as merely an artifact of our lacking dataset. Whatever the case, future studies should assess this possible difference in quantity discrimination abilities, especially when higher numbers are used. Were wolves to be less capable than dogs to distinguish quantities when higher numbers are used, it would put in jeopardy the conclusions from previous studies that compared numerical competence between dogs and wolves.

Nonetheless, it appears that both species may have used non-numerical cues in conjunction with the numerical information to solve this task. For instance, both groups in both species seemed to have had a clear preference for the side with the biggest dot, and a higher probability of failure when that was not the correct side. This bias was not prevalent in all trials, however, having a lesser impact in probe trials (especially for wolves, which did not show the bias in these trials). Nevertheless, this effect may have been strong enough to dampen the influence of the ratio, with generally fewer differences in performance per ratio in trials that controlled for the side with the biggest dot (see the Supplementary Material). It’s possible that our individuals found the larger dot to be a more conspicuous stimulus, and thus had a tendency toward selecting that over the other stimulus with random sizes. Studies in different species have found similar biases toward choosing the largest stimulus, and it has been suggested that this bias may be adaptative in the wild, as choosing the largest pieces of food does usually provide the animal with the most amount of food (Boysen et al., 2001; Beran et al., 2008) although this pattern has not been found in dogs (Miletto Petrazzini and Wynne, 2016). However, given the uneven manifestation of this bias across the conditions and species, it appears to be an artifact of the current setting rather than a phenomenon with real ecological significance. Further research with different paradigms would be needed to determine which of the two possibilities does apply in this case.

More importantly, however, the group trained to choose the smallest amount did select the side with the largest dots and the one with the shapes meant to emulate the negative space between the large dots significantly more often in the control phase, which would be consistent with them choosing based on the appearance of the stimuli rather than the number of dots. That is to say, as the total area covered by the dots had to remain constant between both stimuli, it could be that dogs in group 2 were consistently choosing the side with the biggest dots on average as a rule of thumb to select the side with the lowest number of dots. In dogs, the opposite pattern was not present in group 1 (as the individuals from this group did not choose the star-like shape over the triangular one, nor the smaller dots over the large ones) but wolves trained to select the stimulus with the largest amount of dots did select the stimulus with the smallest ones in the control trials, which would provide credence to them picking the side with the smallest dots regardless of any numerical information. However, since we did not present the two shape control stimuli to the subjects before training them to solve the quantity discrimination task, it remains unclear whether spontaneous preference toward any of the stimuli played a role in their choices, and if it did, to which degree.

Nevertheless, in dogs, the performance of both groups was the same in trials with pairs differing in just one number (the ones with less variation in the average area of the dots between the stimuli). This would imply that, although individuals in group 2 did use alternative cues to solve the task, they also relied on numerical data when that information was not clear enough.

In wolves, however, we did find an effect of the interaction between ratio and group in these trials, with individuals from group 1 performing better at higher ratios than at lower ones (see Supplementary Figure 2 in the Supplementary Material). This does seem to imply that wolves from the first group relied more heavily on non-numerical cues (maybe even exclusively), as their performance was somewhat altered when those cues were not as readily available.

Interestingly, there is no apparent effect of the ratio for these trials with combinations differing on just one number for either of the species. Ward and Smuts (2007) also found a different performance in dogs whenever pairs differing on just one number (i.e., a disparity of one) were presented to the subjects (as they were not able to make the correct choice regardless of the ratio), so it is possible that the low disparity of the numbers used may have dampened the effect of the ratio on the subjects’ success. Future studies should look further into the influence of this factor in quantity discrimination tasks.

The fact that the group trained to choose the smallest amount seemed to rely more on non-numerical information (especially in the case of dogs) could be related to the results of other studies about pattern discrimination in animals. In most studies in which two groups of animals are trained to select different options depending on their magnitude (be it quantity, odor intensity, auditory intensity, etc.), the group trained to choose the higher magnitude seems to have an easier time acquiring the skill (Zielinski and Jakubowska, 1977; Pelz et al., 1997; Watanabe, 1998; Vonk and Beran, 2012; see Inman and Pearce, 2018 for a review).

All in all, it seems like the size of the dots may have played an important role in directing the dogs’ and wolves’ choices. Studies have shown that other species such as fish (Agrillo et al., 2008; Xiong et al., 2018), salamanders (Krusche et al., 2010), cats (Pisa and Agrillo, 2009), and monkeys (Stevens et al., 2007) use mainly non-numerical cues when available, so it should come as no surprise that at least some of our subjects used continuous cues as an aid to solve the task as well (regardless of our efforts in limiting their presence). This could point toward quantity discrimination being an overall more demanding cognitive ability, driving animals to use alternative cues if at all present.

These studies have shown that, although both dogs and wolves are able to distinguish quantities of different ratios and magnitudes, they have a preference toward using non-numerical cues when available. Future studies on canid numerosity should control for non-numerical information more thoroughly (e.g., by having a variable cumulated surface area: making all items presented have the same size on both stimuli in some trials and alternating the side with highest cumulated surface area in others). Furthermore, it would also be interesting to pinpoint, which perceptual cues (density, convex hull, surface area…) allow the animals to make approximations of quantity, and whether they use that information alone in the wild or there is some *sensu stricto* quantity discrimination involved.

## Data Availability Statement

All datasets generated for this study are included in the article/Supplementary Material.

## Ethics Statement

Ethical review and approval was not required for the animal study because no special permission for use of animals (wolves and dogs) in such socio-cognitive studies is required in Austria (Tierversuchsgesetz 2012–TVG 2012). The relevant committee that allows running research without special permissions regarding animals is: Tierversuchskommission am Bundesministerium für Wissenschaft und Forschung (Austria). For all domesticated subjects used, written informed consent was obtained from the owners for the participation of their animals in this study.

## Author Contributions

FR acquired the funding. FR and I-MP designed the study. I-MP and MH conducted the experiments. DR-B and RD managed the data. DR-B, MH, and FR took care of the data analysis and its interpretation. The manuscript was written by DR-B, I-MP, and RD, and revised by FR. All authors read and approved the contents of this manuscript.

## Conflict of Interest

The authors declare that the research was conducted in the absence of any commercial or financial relationships that could be construed as a potential conflict of interest.

## Footnotes

1 The number 8 was used mostly on “small” number pairs, but one pair of “large” numbers does contain it (namely, 8:24; see Table 1). This was due to the relative lack of number pairs with low ratios within the large numbers group.
