# Supplementary material for: Wolves and Dogs May Rely on Non-numerical Cues in Quantity Discrimination Tasks When Given the Choice
Source: Front Psychol. 2020 Sep 11;11:573317. doi: 10.3389/fpsyg.2020.573317 (PMC7518719; doi:10.3389/fpsyg.2020.573317)
Supplement: Supplementary file 1 [file Data_Sheet_1.docx]

Supplementary Material

## Supplementary tables and figures

### Tables

**Supplementary Table 1:** List of dogs that participated in study 1.

|  | **Sex** | **Breed** | **Group** | **Age**  **(years)** | **Previous touch screen experience** | **Date at P1 training** | **Date at P1** | **Date at P2 training** | **Date at P2** | **Date at control** |
| --- | --- | --- | --- | --- | --- | --- | --- | --- | --- | --- |
| **Toffee** | f | Golden Retriever | 1 | 7.00 | Yes | May-Jul11 | Jul-Aug11 | Aug11 | Aug-Sep11 | Sep11 |
| **Chilly** | f | Jack Russell Terrier | 1 | 8.50 | Yes | Jul-Aug11 |  |  |  |  |
| **Flora** | f | Cairn Terrier | 1 | 8.00 | Yes | Aug-Sep11 |  |  |  |  |
| **Ginger** | f | Pug | 1 | 2.00 | Yes | May-Sep11 | Sep-Oct11 |  |  | Oct11 |
| **Aeden** | m | Border Collie | 1 | 3.25 | Yes | Jul-Aug11 | Aug-Sep11 |  |  | Sep11 |
| **Michel** | m | Mixed-breed | 1 | 7.25 | Yes | Oct12 -May13^†^ | | | | May14^†^ |
| **Luna** | f | Siberian Husky | 1 | 1.42 | Yes | ‡ | May-Aug14 |  |  | Aug14 |
| **Talie** | m | Siberian Husky | 1 | 2.92 | Yes | ‡ | May-Aug14 |  |  | Aug14 |
| **Miley** | f | Border Collie | 1 | 1.08 | No | Jun11 | Jun-Aug11 |  |  | Sep11 |
| **Havanna** | f | Beagle | 2 | 5.33 | Yes | Jul-Aug11 |  |  |  |  |
| **Ida** | f | German Shepherd | 2 | 3.83 | Yes | May-Jun11 | Jul-Sep11 |  |  | Sep11 |
| **Bertl** | m | Australian Shepherd | 2 | 8.08 | Yes | May-Jul11 | Jul-Sep11 |  |  | Sep11 |
| **Guinness** | f | Border Collie | 2 | 6.42 | Yes | May-Jun11 | Jun-Aug11 | Aug-Sep11 | Sep-Oct11 |  |
| **Caya** | f | Border Collie | 2 | 3.58 | Yes | Jun; Aug11 | Aug-Sep11 |  |  | Sep11 |
| **Xela** | f | Border Collie | 2 | 3.92 | Yes | Jun-Jul11 | Jul-Aug11 | Aug-Sep11 | Sep11 | Oct11 |
| **Chio** | f | Border Collie | 2 | 2.08 | Yes | Aug11 | Aug-Sep11 |  |  | Sep11 |
| **Oszkar** | m | Mixed-breed | 2 | 9.17 | Yes | Apr-Jul12 | Jul-Dec12 |  |  |  |
| **Tyrell** | m | Golden Retriever | 2 | 2.67 | No | Aug11 | Aug-Sep11 |  |  | Sep11 |
| **Shiloh** | f | Pug | 2 | 1.92 | No | Aug-Sep11 |  |  |  |  |
| **Banja** | f | Mixed-breed | 2 | 1.75 | No | Jul-Aug11 | Aug-Sep11 | Sep11 | Sep-Oct11 | Oct-Nov11 |

^†^: The date in which these sessions were carried out was corrupted on the output files, and thus it became impossible to retrieve it. It is also possible that the dates presented may be incorrect as well.
‡: The output files for these sessions were lost, and thus it is not possible to determine the dates in which they took place.

**Supplementary Table 2:** Wolves that participated in study 2. As the duration of this study was much longer than study 1, ages of the subjects are presented as an average between the age at the first and last trial performed. The semicolons are used to separate session over periods in which experimenting did not take place (due to malfunction of the apparatus). All subjects received additional training sessions (for the corresponding phase of the study) after every of such breaks.

|  | **Sex** | **Group** | **Average age (years)** | **Date at P1 training** | **Date at P1** | **Date at P2 training** | **Date at P2** | **Date at control** |
| --- | --- | --- | --- | --- | --- | --- | --- | --- |
| **Chitto** | m | 1 | 2.88 | Feb-May13 | Jun-Nov13 | Dec13–May14 | May14; Jan-May15; Nov15 | Dec15 |
| **Kaspar** | m | 1 | 4.42 | Jul-Sep11 | Sep-Dec11 | Jan12-May13 | Jul-Nov13 | Nov13 |
| **Shima** | f | 1 | 4.63 | Jun-Aug11 | Aug-Oct11 | Nov11-Jan14 | Jan-Jun14; Dec14-Feb15 | Mar-Apr15 |
| **Tatonga** | f | 1 | 3.50 | Jul-Aug12 | Aug12-Feb13 | Feb-Dec13^†^ |  |  |
| **Geronimo** | m | 1 | 4.38 | Feb13-May14 |  |  |  |  |
| **Amarok** | m | 2 | 3.33 | Sep13-Mar14 | Mar-May14; Jan-May15; Nov-Dec15 | Jan-Apr16; Jul-Oct16 | Oct-Dec16 | Dec16-Jan17 |
| **Aragorn** | m | 2 | 3.71 | Jul-Aug11 | Aug-Nov11 | Nov11-Feb12 | Feb-Jun12 | Jun-Jul12 |
| **Kay** | f | 2 | 1.29 | Feb-Mar13 | Mar-Oct13 | Oct-Nov13^†^ |  |  |
| **Nanuk** | m | 2 | 5.67 | Feb13 | Feb-Sep13 | Sep13-Jan14 | Jan-Jun14; Jan-Apr15 | Apr16; Oct-Dec16 |
| **Una** | f | 2 | 3.29 | Nov13-Mar14 | Apr-May14;  Mar-May15; Nov15-Jan16; Apr16; Aug-Dec16 |  |  |  |
| **Yukon** | f | 2 | 4.38 | Feb13-Jun14 |  |  |  |  |

^†^: Sessions interrupted due to passing away of the subject.

**Supplementary Table 3:** Sessions performed and data availability for the wolves. **(A)** Phase 1; **(B)** Phase 2.

**(A)**

|  | **Phase 1** | | | | | | | | | | | | | | | | | | | | | | | | | | | | | |  |  |
| --- | --- | --- | --- | --- | --- | --- | --- | --- | --- | --- | --- | --- | --- | --- | --- | --- | --- | --- | --- | --- | --- | --- | --- | --- | --- | --- | --- | --- | --- | --- | --- | --- |
|  | **Training** | **Level 1** | | | | | | **Level 2** | | | | | **Level 3** | | | | **Level 4** | | | | | | **Level 5** | | | | **Level 6** | | | |  |  |
|  |  | 1 | 2 | 3 | 4 | 5 | 6 | 1 | 2 | 3 | 4 | 5 | 1 | 2 | 3 | 4 | 1 | 2 | 3 | 4 | 5 | 6 | 1 | 2 | 3 | 4 | 1 | 2 | 3 | 4 |  |  |
| **Amarok** | x | x | x | x | x |  |  | x | x | x | x |  | x | x | x | x | x | x | x | x |  |  | x | x | x | x | x | x | x | x |  |  |
| **Aragorn** | x | x | x | x | x |  |  | x | x | x | x | x | x | x | x | x | x | x | x | x | x | x | - | - | x | x | x | x | x | x |  |  |
| **Chitto** | x | x | x | x | x |  |  | x | x | x | x |  | x | x | x | x | x | x | x | x |  |  | x | x | x | x | x | x | x | x |  |  |
| **Geronimo** | x |  |  |  |  |  |  |  |  |  |  |  |  |  |  |  |  |  |  |  |  |  |  |  |  |  |  |  |  |  |  |  |
| **Kaspar** | x | x | x | x | x | x | x | x | x | x | x |  | x | x | x | x | x | x | x | x |  |  | x | x | x | x | x | x | x | x |  |  |
| **Kay** | x | x | x | x | x |  |  | x | x | x | x | x | x | x | x | x | x | x | x | x |  |  | x | x | x | x | x | x | x | x |  |  |
| **Nanuk** | x | x | x | x | x |  |  | x | x | x | x |  | x | x | x | x | x | x | x | x |  |  | x | x | x | x | x | x | x | x |  |  |
| **Shima** | x | x | x | x | x |  |  | x | x | x | x |  | x | x | x | x | x | x | x | x | x |  | x | x | x | x | x | x | x | x |  |  |
| **Tatonga** | x | x | x | x | x |  |  | x | x | x | x | x | x | x | x | x | x | x | x | x |  |  | x | x | x | x | x | x | x | x |  |  |
| **Una** | x | - | x | x | x |  |  | x | x | x | x | x | x | x | x | x | x | x | x | x |  |  | x | x | x | x |  |  |  |  |  |  |
| **Yukon** | x |  |  |  |  |  |  |  |  |  |  |  |  |  |  |  |  |  |  |  |  |  |  |  |  |  |  |  |  |  |  |  |
|  |  |  |  |  |  |  |  |  |  |  |  |  |  |  |  |  |  |  |  |  |  |  |  |  |  |  |  |  |  |  |  |  |

**(B)**

|  | **Phase 2** | | | | | | | | | | | | | | | | | | | | | | | | | | | | |  | | | | | | |
| --- | --- | --- | --- | --- | --- | --- | --- | --- | --- | --- | --- | --- | --- | --- | --- | --- | --- | --- | --- | --- | --- | --- | --- | --- | --- | --- | --- | --- | --- | --- | --- | --- | --- | --- | --- | --- |
|  | **Training** | **Level 1** | | | | **Level 2** | | | | **Level 3** | | | | | | | **Level 4** | | | | **Level 5** | | | | **Level 6** | | | | | **Control** | | | | | | |
|  |  | 1 | 2 | 3 | 4 | 1 | 2 | 3 | 4 | 1 | 2 | 3 | 4 | 5 | 6 | 7 | 1 | 2 | 3 | 4 | 1 | 2 | 3 | 4 | 1 | 2 | 3 | 4 | 5 | 1 | 2 | 3 | 4 | 5 | 6 | 7 |
| **Amarok** | x | x | x | x | x | x | x | x | x | x | x | x | x |  |  |  | x | x | x | x | x | x | x | x | x | x | x | x |  | x | x | x | x | x | x | x |
| **Aragorn** | x | x | x | x | x | - | - | - | - | - | x | x | x |  |  |  | x | x | x | x | x | x | x | x | x | x | x | x | x | x | x | x | x | x | x | x |
| **Chitto** | x | x | x | x | x | x | x | x | x | x | x | x | x |  |  |  | x | x | x | x | x | x | x | x | x | x | x | x |  | x | x | x | x | x | x | x |
| **Kaspar** | x | x | x | x | x | x | x | x | x | x | x | x | x |  |  |  | x | x | x | - | x | x | x | x | x | x | x | x |  | x | x | x | x | x | x |  |
| **Kay** | x |  |  |  |  |  |  |  |  |  |  |  |  |  |  |  |  |  |  |  |  |  |  |  |  |  |  |  |  |  |  |  |  |  |  |  |
| **Nanuk** | x | x | x | x | x | x | x | x | x | x | x | x | x | x | x | x | x | x | x | x | x | x | x | x | x | x | x | x |  | x | x | x | x | x | x |  |
| **Shima** | x | x | x | x | x | x | x | x | x | x | x | x | x | x |  |  | x | x | x | x | x | x | x | x | x | x | x | x |  | x | x | x | x | x | x |  |
| **Tatonga** | x |  |  |  |  |  |  |  |  |  |  |  |  |  |  |  |  |  |  |  |  |  |  |  |  |  |  |  |  |  |  |  |  |  |  |  |

**"x":** Sessions performed; **"-":** Sessions not performed; **red:** Sessions done in excess/not carried out due to human error; **yellow:** Sessions performed, but data missing; **blue:** Sessions performed, data available

### Figures


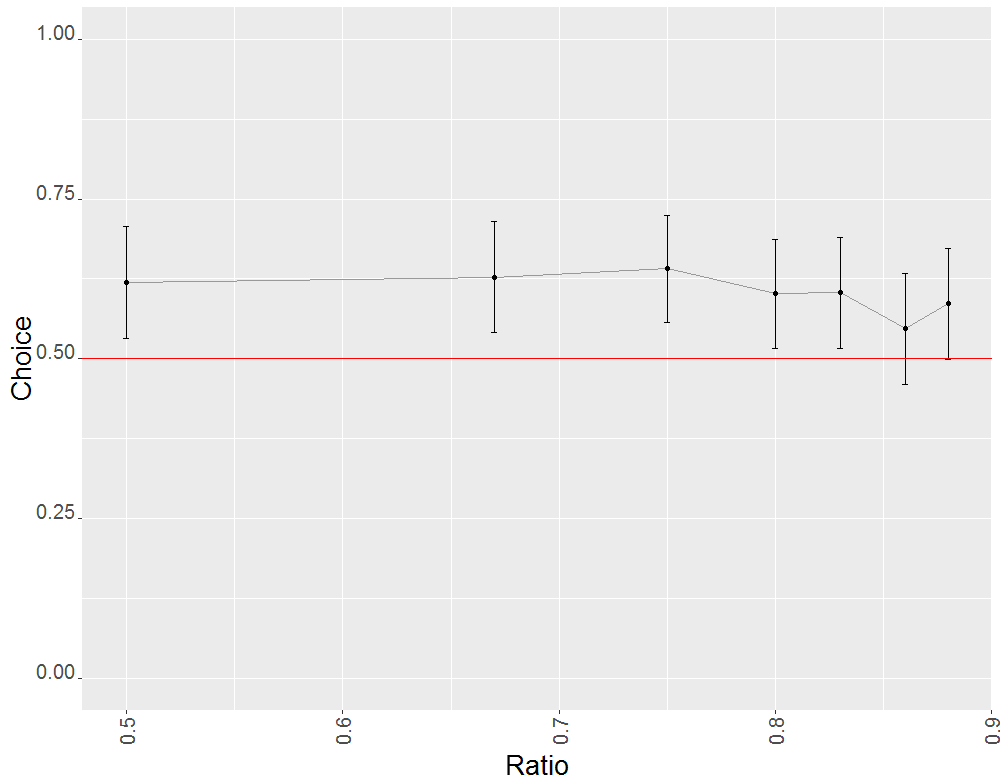


**Supplementary Figure 1:** Effect of the ratio in the trials differing in just one number in study 1 (dogs). Error bars set at the 95% confidence interval; the red line represents probability of success by choosing at random.


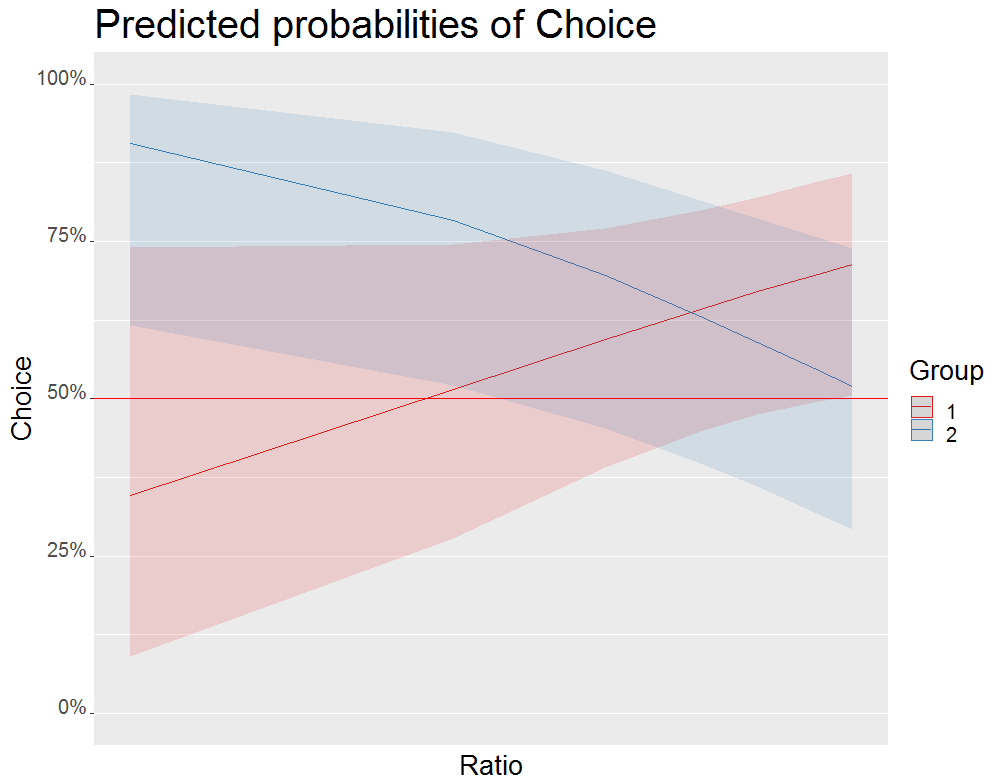


**Supplementary Figure 2:** Interaction between ratio and group in the trials of number pairs differing only in one number for study 2 (wolves).


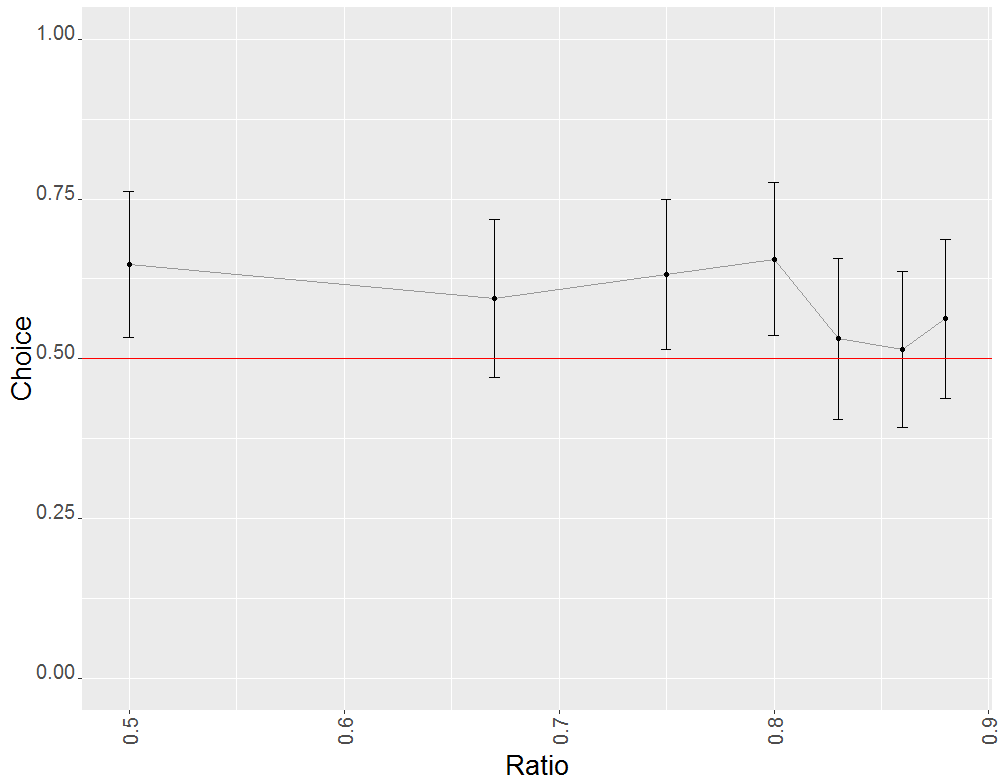


**Supplementary Figure 3:** Effect of the ratio in the trials differing in just one number in study 2 (wolves). Error bars set at the 95% confidence interval; the red line represents probability of success by choosing at random.

## Pre-training

Touch screen inexperienced dogs (n= 13) had to first learn to approach the screen and press it to get a reward. During the first steps of shaping, any kind of behavior directed towards the touch screen (looking, walking towards it...) would be rewarded with a piece of food. In later stages of shaping, the subject would have to touch the touch screen to receive a reward. When the subjects consistently touched the touch screen during the shaping sessions, they were trained on the pre-training task, in which they learned to discriminate between two geometrical forms (some of the subjects were rewarded when they pressed the side of the screen with a circle and the others when they pressed the side with a square).

In contrast, the touch screen experienced dogs (n= 16) had worked in the past with a different touch screen that would react when touched softly. Thus, these dogs had to learn that more pressure was needed with this particular apparatus, and that treats fell out of a machine behind them instead of underneath the screen (as was the case in the previous experiment in which they participated). As such, these dogs performed the geometrical form discrimination task as well.

Touch screen inexperienced dogs could proceed with the training phase as soon as they made 20 out of 30 correct first choices in 4 out of 5 sessions. The experienced dogs, however, had to reach 20 out of 30 correct choices within one session since they only needed to become comfortable with the new set-up.

All wolves tested had previous experience with the touch screen after being tested for another study (currently unpublished). Before that study took place, they also performed the geometrical form discrimination task in the same manner as the dogs, with a criterion of 20 out of 30 correct first choices in 4 out of 5 sessions.

Duration of the pre-training varied drastically between the subjects, with some achieving learning criterion in the minimum amount of sessions (five) and some others requiring tens of sessions to acquire the skill.

## Additional data analyses

Aside from the effects discussed in the results section for both studies, we found an effect of the interaction between the ratio and the combination of stimuli in the rewarded trials of both phases (Phase 1, dogs: Wald χ^2^=28.023, p<0.001; Phase 2, dogs: Wald χ^2^=20.646, p<0.001; Phase 1, wolves: Wald χ^2^=14.387, p<0.001; Phase 2, wolves: Wald χ^2^=8.403, p=0.015). In both cases, the effects of the ratio were less prevalent in the trials with the biggest dot/random combination rather than in the one/cumulated one; but not than the smallest dot/random combination; phase 1, dogs: biggest dot/random vs. one/cumulated z=-5.247, p<0.001, biggest dot/random vs. smallest dot/random, z=-1.676, p=0.094; phase 2, dogs: biggest dot/random vs. one/cumulated z=-4.543, p<0.001, biggest dot/random vs. smallest dot/random, z=-1.745, p=0.081; phase 1, wolves: biggest dot/random vs. one/cumulated z=-3.673, p<0.001, biggest dot/random vs. smallest dot/random, z-0.664, p=0.507; phase 2, wolves: biggest dot/random vs. one/cumulated z=-2.479, p=0.013, biggest dot/random vs. smallest dot/random, z=0.265, p=0.791 (see Figures S4 and S5).

Additionally, in dogs, we found an effect of the interaction between group and combination of stimuli in the rewarded trials in phase 2 (Wald χ^2^=6.809, p=0.033). However, there were no significant differences between the different levels and groups (although the combination one/cumulated showed a tendency towards poorer performance in group 2; GLMM: z=-1.884, p=0.060).

It is interesting to note that the effect of variables other than ratio seems more pronounced in rewarded trials. In wolves, the effect of the combination of stimuli was only present in rewarded trials, and out of the dogs' probe trials, only the ones in the first phase were affected. Furthermore, the compounded effects here introduced are exclusive to the rewarded trials, for both phases and both species. This may be explained by a ceiling effect: as the animals were thoroughly trained for these combinations, success per ratio was maximized, which led to other features of the stimuli, inclinations of the subjects, and the training they received to bear a larger impact on the results.


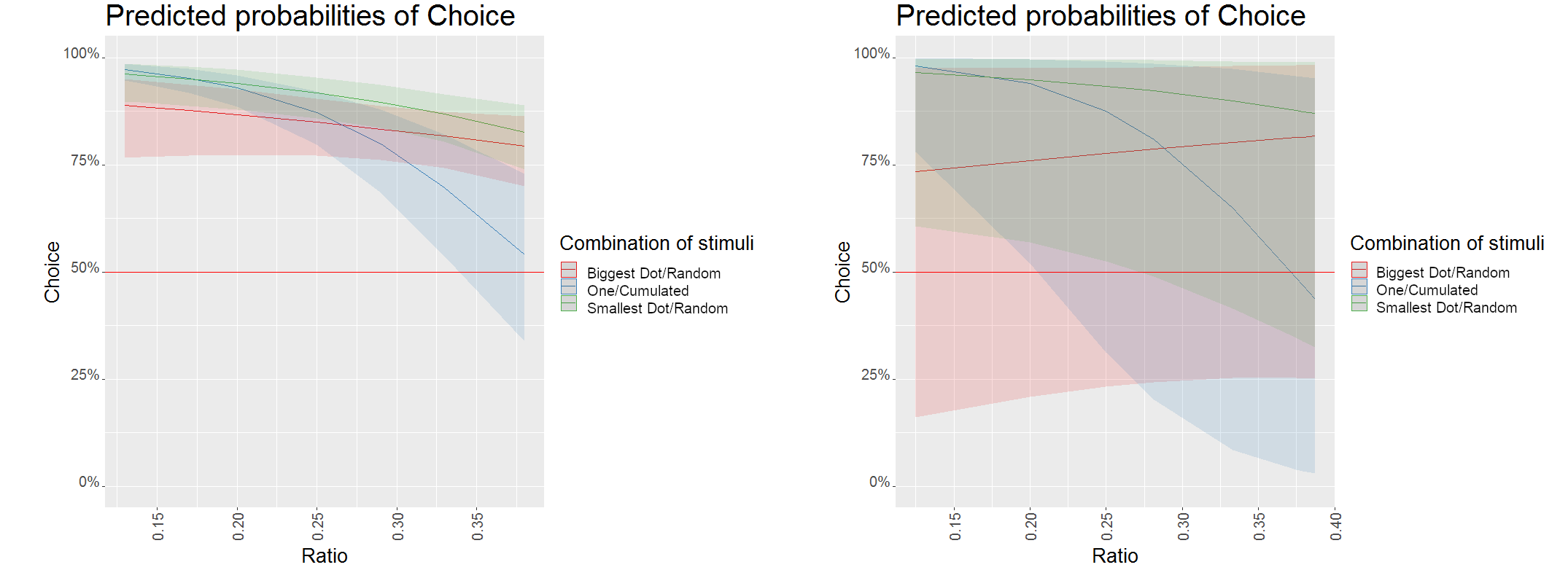


**(A)**


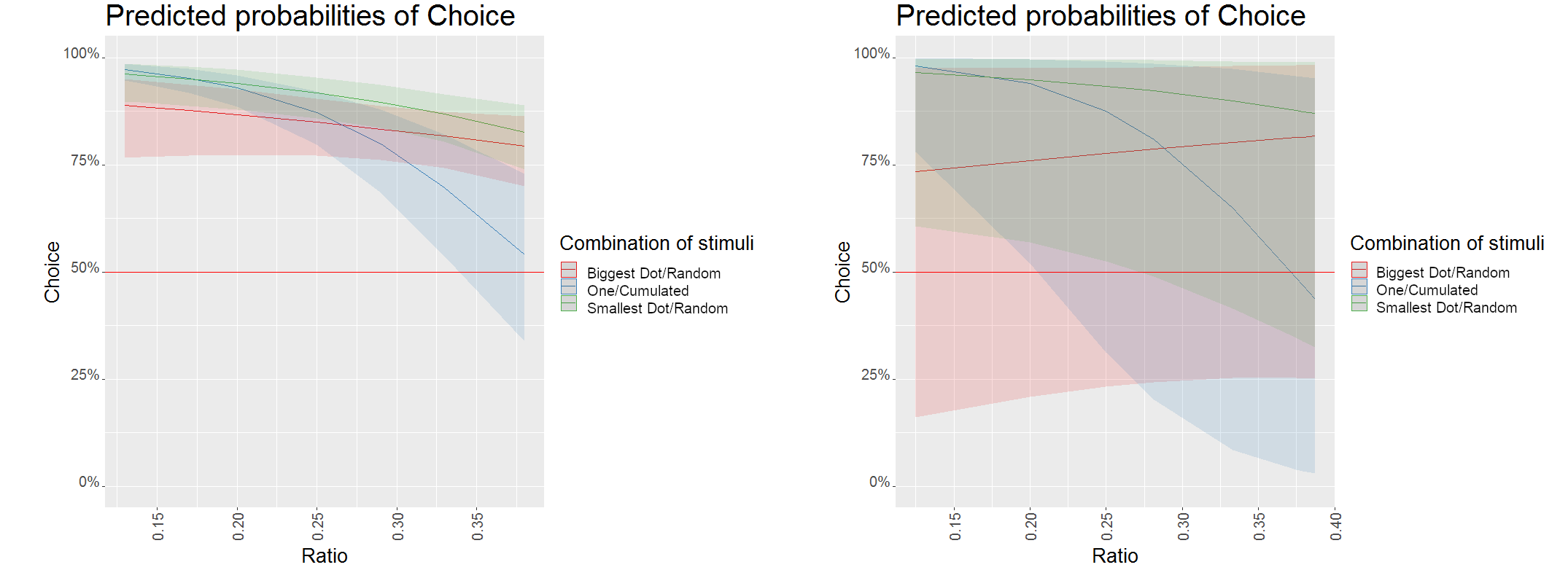


**(B)**

**Supplementary Figure 4:** Combined effect of the ratio and the combination of stimuli over the probability of success for rewarded trials of **(A)** phase 1 and **(B)** phase 2 in dogs.


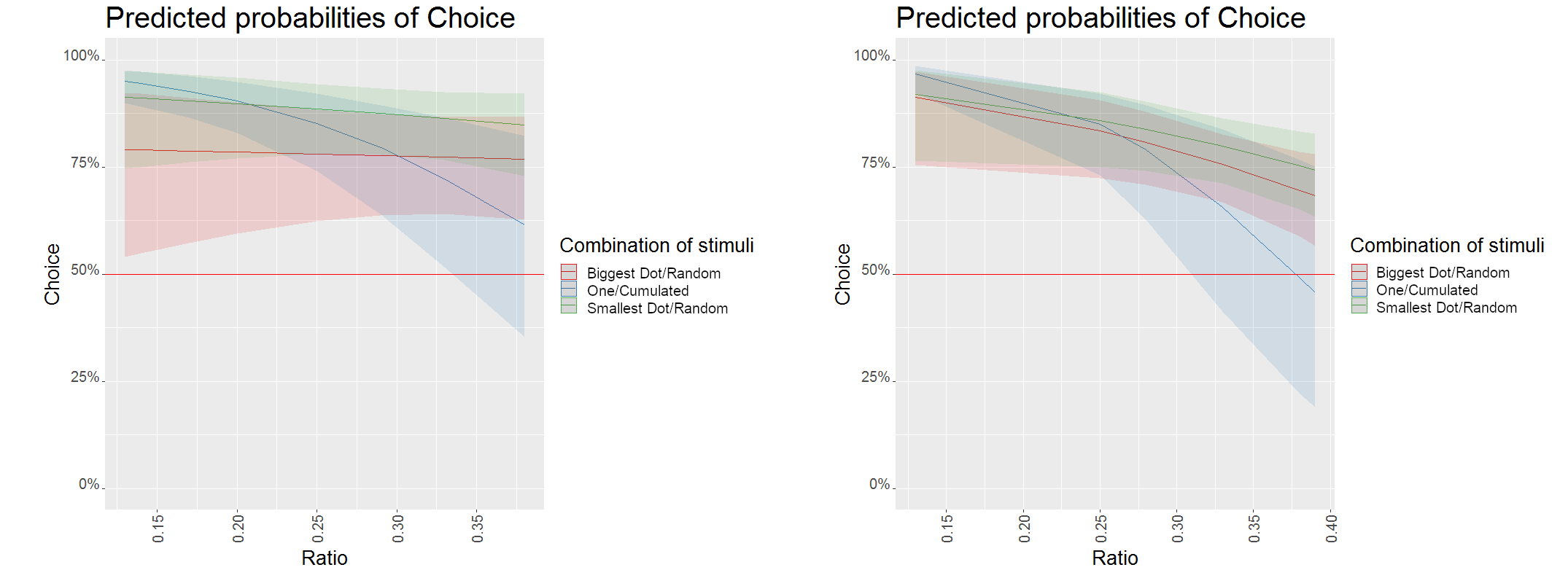


**(A)**


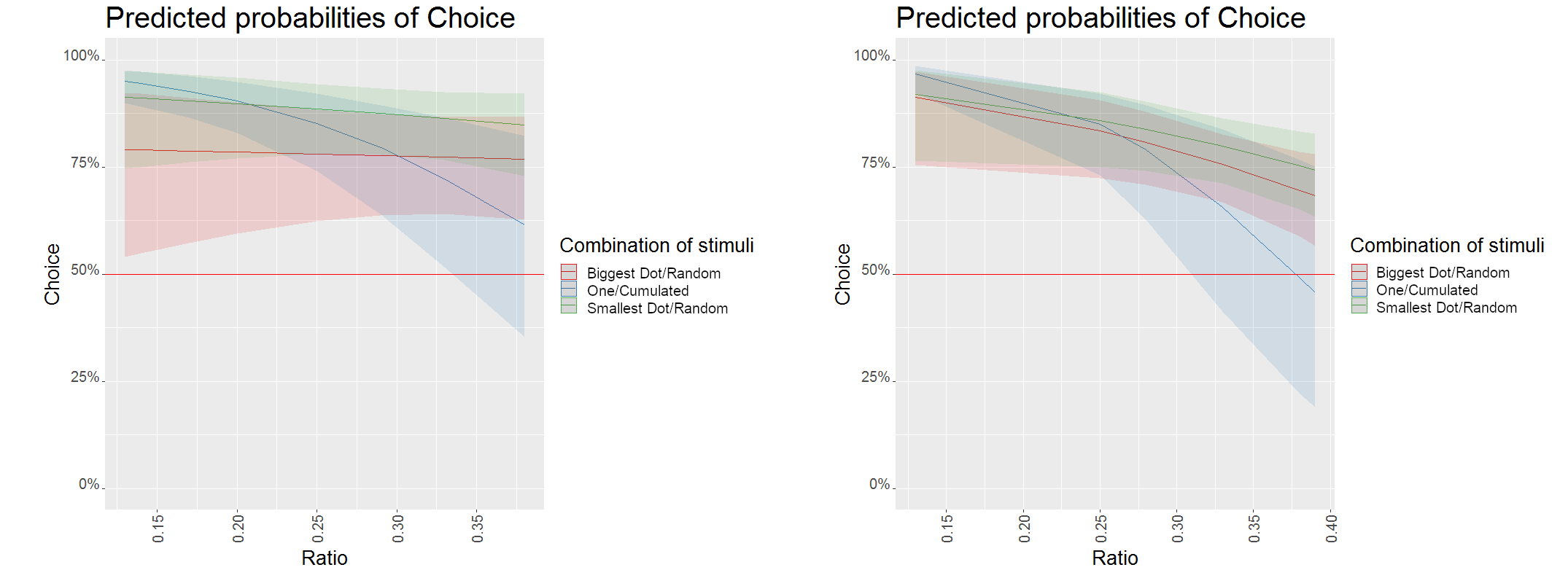


**(B)**

**Supplementary Figure 5:** Combined effect of the ratio and the combination of stimuli over the probability of success for rewarded trials of *a)* phase 1 and *b)* phase 2 in wolves.
